# Facilitating uniform lithium-ion transport via polymer-assisted formation of unique interfaces to achieve a stable 4.7 V Li metal battery

**DOI:** 10.1093/nsr/nwaf182

**Published:** 2025-05-10

**Authors:** Xinqi Li, Zhaojie Li, Chuang Li, Fei Tian, Zhengping Qiao, Danni Lei, Chengxin Wang

**Affiliations:** State Key Laboratory of Optoelectronic Materials and Technologies, School of Materials Science and Engineering, Sun Yat-Sen University, Guangzhou 510275, China; State Key Laboratory of Optoelectronic Materials and Technologies, School of Materials Science and Engineering, Sun Yat-Sen University, Guangzhou 510275, China; State Key Laboratory of Optoelectronic Materials and Technologies, School of Materials Science and Engineering, Sun Yat-Sen University, Guangzhou 510275, China; State Key Laboratory of Optoelectronic Materials and Technologies, School of Materials Science and Engineering, Sun Yat-Sen University, Guangzhou 510275, China; State Key Laboratory of Optoelectronic Materials and Technologies, School of Materials Science and Engineering, Sun Yat-Sen University, Guangzhou 510275, China; State Key Laboratory of Optoelectronic Materials and Technologies, School of Materials Science and Engineering, Sun Yat-Sen University, Guangzhou 510275, China; State Key Laboratory of Optoelectronic Materials and Technologies, School of Materials Science and Engineering, Sun Yat-Sen University, Guangzhou 510275, China

**Keywords:** lithium metal batteries, electrolyte additives, Al(EtO)_3_ nanowires, high-voltage cycling, uniform lithium-ion flow

## Abstract

Achieving stable cycling of lithium metal batteries (LMBs) at high voltages presents a significant challenge due to interfacial instability and uneven lithium-ion transport, leading to dendrite formation and cathode degradation. Constructing a solid-electrolyte interphase (SEI) that facilitates fast and uniform ion transport is crucial for enhancing the stability of electrode structures. However, current research mainly focuses on interfacial instability while neglecting uneven ion transport, which is even more critical. In this study, we develop a novel electrolyte system, PAFE, by incorporating aluminum ethoxide (Al(EtO)_3_), fluoroethylene carbonate (FEC), and pentafluorocyclotriphosphazene (PFPN) into a carbonate-based electrolyte. Al(EtO)_3_ serves as a crosslinking agent, facilitating the formation of a three-dimensional polymer network that promotes the uniform deposition of inorganic components such as LiF, Li_3_N, Li_3_P and Al_2_O_3_ within the SEI and cathode-electrolyte interphase (CEI). These uniform interphases lower the activation energy for lithium-ion transport, thereby ensuring consistent ion flow and reducing internal stress within the electrodes. As a result, Li||LiNi_0.8_Co_0.1_Mn_0.1_O_2_ (NCM811) cells with PAFE exhibit exceptional cycling stability, retaining 80% capacity over 140 cycles at a high cut-off voltage of 4.7 V. Furthermore, 1 Ah pouch cells demonstrate excellent cycling performance, highlighting the potential of this electrolyte system for practical high-energy-density LMB applications.

## Teaser

This study introduces a novel electrolyte that enhances interfacial stability and promotes ion transport uniformity to reduce internal stress and strain within electrodes, thereby enabling lithium metal batteries with long cycle life, high energy density, and non-flammable properties.

## INTRODUCTION

Lithium metal anodes (LMAs), with a high theoretical capacity of 3860 mAh g^−1^ and the lowest electrochemical potential, are considered ideal candidates for replacing graphite anodes in lithium batteries [1]. When lithium metal batteries (LMBs) are paired with high-capacity nickel-rich cathodes (NCM) and operated at elevated charging cut-off voltages, their energy density increases significantly [2,3]. However, when operating voltages exceed 4.4 V, the electrode-electrolyte interface becomes highly unstable. Under such high-voltage conditions, liquid electrolytes become increasingly susceptible to oxidative degradation. This triggers the formation of unstable, non-uniform interfacial layers that disrupt ionic-flux uniformity. The ensuing uneven Li-ion distribution amplifies localized internal stress within the electrodes, further compromising structural integrity during repeated cycling [4,5]. On the cathode side, deep delithiation triggers abrupt changes in lattice parameters (the H2→H3 phase transition), which result in anisotropic contraction of the layered structure and the generation of localized stress [6,7]. Spatial differences in the rate of lithium ion deintercalation can significantly amplify this mechanical stress; regions undergoing rapid delithiation develop shear stresses due to disparities in lattice contraction rates [8]. This concentration of stress preferentially induces microcrack formation along grain boundaries, promoting electrolyte penetration and initiating adverse side reactions that accelerate capacity fading and increase impedance [8,9]. On the anode side, inhomogeneous Li-ion concentration leads to irregular Li deposition and dendrite formation that can penetrate the solid-electrolyte interphase (SEI) and separator, causing internal short-circuits and elevating the risk of thermal runaway [10,11]. While all-solid-state electrolytes theoretically address these issues by completely eliminating liquid decomposition pathways [12], their practical implementation faces fundamental limitations: insufficient ionic conductivity, exacerbated interfacial resistance from poor solid-solid contact, and inadequate rate capability for high-energy applications [13]. Consequently, advanced liquid electrolyte engineering remains essential for synchronizing the stability of SEI and cathode-electrolyte interphase (CEI) layers with uniform ion transport properties. Key design strategies must focus on tailoring interfacial layer composition and distribution to achieve these objectives.

Controlling electrolyte composition through additives is an effective strategy to enhance SEI and CEI properties [14,15]. Additives such as fluoroethylene carbonate (FEC) and pentafluorocyclotriphosphazene (PFPN) have been employed to enrich the interphases with inorganic ionic conductors such as LiF, Li_3_N and Li_3_P, thereby improving ionic conductivity and interfacial stability [16,17]. However, these additives usually generate interphase components through simple chemical decomposition, yielding uneven distributions that mirror local surface variations [18,19]. Modifying cathodes with Al_2_O_3_ has been shown to enhance structural stability [20]. However, commonly used techniques such as atomic layer deposition face challenges in controlling layer thickness and uniformity, and are not easily scalable [21]. To address these challenges, we propose incorporating aluminum ethoxide (Al(EtO)_3_) into the electrolyte as a cross-linking agent to form a three-dimensional polymer network with FEC and PFPN. This strategy aims to achieve a uniform distribution of inorganic components within the SEI and CEI, thereby enhancing lithium-ion (Li^+^) transport and reducing internal stress within the electrodes.

In this study, we introduce a novel non-aqueous electrolyte (PAFE) by incorporating synthesized Al(EtO)_3_ nanowires into a LiPF_6_-based carbonate electrolyte containing FEC and PFPN. Al(EtO)_3_ serves as a cross-linking agent, facilitating the formation of a three-dimensional polymer network with FEC and PFPN. This network promotes the homogeneous deposition of inorganic components such as LiF, Li_3_N, Li_3_P and Al_2_O_3_, enhancing the mechanical strength and stability of the SEI and CEI layers. The uniform interfacial layer produced by PAFE markedly reduces Li^+^ diffusion barriers, thereby enabling homogeneous ionic flux across electrode surfaces. This uniformity mitigates structural degradation caused by uneven stress distribution in both electrodes. Additionally, the chemical and thermal stability of Al_2_O_3_ contributes to a robust interfacial layer, enhancing overall electrode integrity [22]. The introduction of PFPN also improves the flame retardancy of the electrolyte, enhancing safety under high-voltage conditions [23,24]. When paired with high-loading commercial LiNi_0.8_Co_0.1_Mn_0.1_O_2_ (NCM811) cathodes and LMAs, PAFE demonstrates exceptional cycling stability, retaining 80% of its capacity after 140 cycles at 4.7 V. Furthermore, under practical conditions, a 1 Ah LMB pouch cell exhibits stable performance over 80 cycles, underscoring its commercial potential.

## RESULTS AND DISSCUSSION

### Design strategy

To overcome the uneven distribution of inorganic components in traditional electrolytes like PFE, we developed PAFE by incorporating Al(EtO)_3_ nanowires. Al(EtO)_3_ acts as a crosslinker, interacting with FEC and PFPN to form a three-dimensional polymer network. This network promotes the uniform co-deposition of inorganic components (e.g. Li_3_N, Li_3_P, LiF and Al_2_O_3_) within the SEI and CEI layers, preventing aggregation and ensuring even distribution.

**Figure 1.**
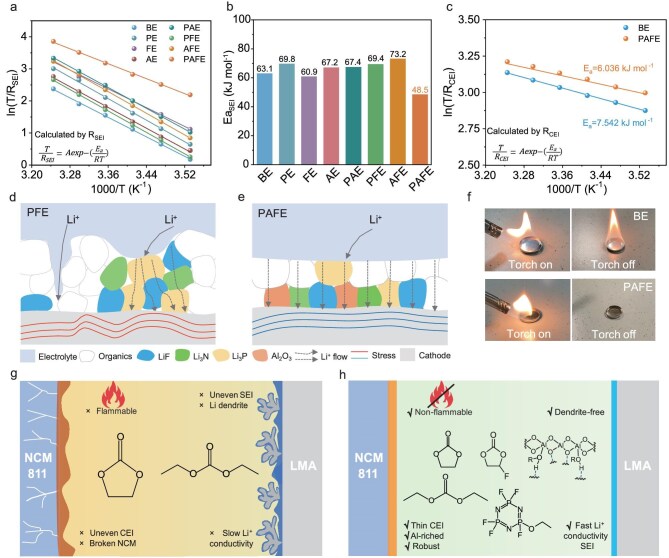
Design strategy. (a) The corresponding Arrhenius behavior and apparent activation energy derived from *R*_SEI_ in Nyquist plots at various temperatures of Li||Li symmetric cells with different electrolytes. All Li||Li cells were cycled for 10 rounds at 1 mA cm^−2^ and 1 mAh cm^−2^ to establish a stable SEI. (b) Comparison of E_aSEI_ values. (c) The corresponding Arrhenius behavior and apparent activation energy derived from *R*_CEI_ in Nyquist plots at various temperatures of NCM||NCM symmetric cells with different electrolytes. Schematics of the interphases formed in PFE (d) and PAFE (e). (f) Flammability tests of BE and PAFE electrolytes. Protection mechanisms of the SEI and CEI formed in BE (g) and PAFE (h).

To quantitatively assess the impact of interface uniformity on Li^+^ transport, we employed temperature-dependent electrochemical impedance spectroscopy (EIS) within the 10–35°C range to measure the activation energy (E_a_) for Li^+^ migration [25]. The EIS curves at different frequencies were fitted using an equivalent circuit model (Fig. S1), providing insights into the SEI resistance (*R*_SEI_). The activation energy represents the energy required to overcome barriers during ion transport at the interface, with lower E_a_ values indicating more efficient ion conduction pathways [26,27]. By systematically testing eight different electrolytes–BE, AE, PE, FE, AFE, PFE, PAE and PAFE, for their E_aSEI_ values (detailed formulations are provided in Table S1, the E_aSEI_ for these electrolytes was deduced via Arrhenius fitting as shown in Fig. S2), we aimed to correlate interface structure with ion transport properties, thereby providing quantitative evidence that uniformly distributed inorganic phases significantly reduce transport barriers. Figure 1a and b illustrates the E_aSEI_ fitting curves and E_aSEI_ values for Li^+^ transport in these eight electrolytes, demonstrating that BE has an E_aSEI_ of 63 kJ mol^−1^, FE is slightly lower at 60 kJ mol^−1^, and PAFE exhibits the lowest E_aSEI_ at only 48 kJ mol^−1^. The E_aSEI_ values for the remaining electrolytes are higher than that of BE. This indicates that PFPN, FEC or Al(EtO)_3_, used individually or in pairs, failed to produce interfaces with lower activation energies. They lack the unique polymer network formed by Al(EtO)_3_ interacting with FEC and PFPN, resulting in an uneven distribution of inorganic phases that hinders ion transport. In contrast, the cross-linked polymer structure of PAFE ensures interface uniformity, significantly lowering the E_aSEI_ and enhancing ion conductivity. These E_aSEI_ test results not only quantify the differences in ion transport efficiency among the various electrolytes but also support our hypothesis that uniformly distributed inorganic components can effectively reduce ion transport barriers, thereby facilitating more efficient ion migration. Furthermore, within the CEI, BE exhibits an E_aCEI_ of 7.5 kJ mol^−1^, whereas PAFE shows 6 kJ mol^−1^, further highlighting the superior ion transport performance of PAFE in the CEI layer (Fig. 1c; E_aCEI_ values were obtained by Arrhenius fitting, as shown in Fig. S3).

In addition to the reduced activation energy, improvements in interface morphology with the PAFE electrolyte are equally crucial. Figure 1d and e illustrates the schematic interfaces formed by PFE and PAFE. The interface formed by PFE exhibits an uneven distribution of inorganic components, leading to irregular Li^+^ transport pathways and localized mechanical stress concentrations. Over time, this non-uniformity can induce microcracks and structural degradation, thereby compromising battery cycle stability. In contrast, the PAFE electrolyte promotes a uniform distribution of inorganic components, ensuring consistent Li^+^ flux across the electrode surface, reducing internal stresses, and thereby enhancing structural integrity and battery performance. Figure 1f presents direct-ignition tests for BE and PAFE electrolytes. BE ignites upon exposure to a flame and continues to burn after the igniter is removed, whereas PAFE does not ignite, indicating superior flame retardancy. The flame retardancy of the PAFE electrolyte primarily comes from PFPN. Upon overheating, PFPN releases phosphorus and fluorine radicals that neutralize hydrogen radicals from the solvent, effectively halting combustion. In addition, its decomposition forms a non-flammable liquid film that covers the electrode surface, isolating it from oxygen, and releases inert gases such as NH_3_ and N_2_ that dilute combustible gases [24]. Figure 1g and h illustrates the schematic of Li||NCM811 battery systems using BE and PAFE electrolytes. PAFE forms a more uniform and stable interface, which mitigates localized stress during cycling and enhances both battery performance and safety at high voltages. Through systematic activation energy measurements and interface morphology analyses, our design strategy has been thoroughly validated. This approach not only effectively addresses the issue of uneven interfaces in traditional electrolytes but also paves the way for the development of high-performance and long-life LMBs.

### Physicochemical characterization of electrolyte solutions

In a 1 M LiPF_6_ solution with ethylene carbonate (EC) and diethyl carbonate (DEC) in a 1:1 volume ratio, we added 20 vol.% FEC, 10 vol.% PFPN and 1 wt.% Al(EtO)_3_ to prepare the PAFE. After 2 hours of ultrasonication, the solution became transparent, indicating complete dissolution of Al(EtO)_3_. In contrast, when 1 wt.% Al(EtO)_3_ was added to BE to obtain AE, the solution remained turbid after ultrasonic treatment, demonstrating that 1 wt.% Al(EtO)_3_ is difficult to dissolve in BE (Fig. S4a). When stored in an argon-filled glovebox at room temperature for 3 days, PAFE exhibited a color change, suggesting chemical interactions (Fig. S4b). To elucidate the reaction mechanisms between Al(EtO)_3_, PFPN and FEC, we prepared and incubated mixtures of Al(EtO)_3_/FEC, Al(EtO)_3_/PFPN, PFPN/FEC and PFPN/Al(EtO)_3_/FEC at 60°C for 5 days under static (non-stirred) conditions to simulate the aging process and promote the coordination reactions among Al(EtO)_3_, PFPN and FEC. Subsequently, we performed Fourier-transform infrared spectroscopy (FTIR) on the supernatants, and solid-state nuclear magnetic resonance (NMR) and gel-permeation chromatography (GPC) on the dried precipitates.

**Figure 2.**
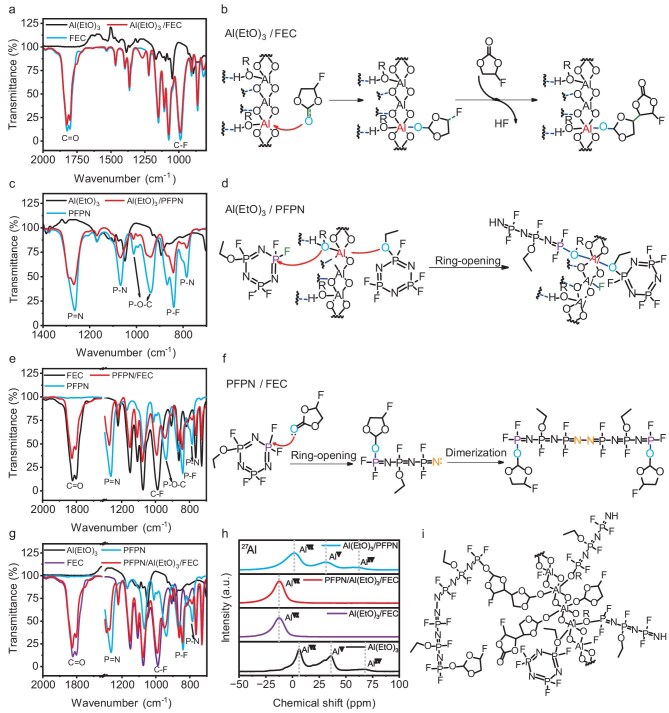
Physicochemical characterization of the electrolyte additives. (a, c, e, g) FTIR spectra of pure Al(EtO)_3_ and precipitates from various solutions. (b, d, f) Proposed reaction mechanisms of Al(EtO)_3_/FEC, Al(EtO)_3_/PFPN and PFPN/FEC mixtures. (h) ^27^Al solid-state NMR spectra. (i) Proposed formation of PFPN/Al(EtO)_3_/FEC complexes.

Figure 2a shows that the FTIR spectrum of the Al(EtO)_3_/FEC sample displays reduced intensities for the C–F and C=O absorption peaks at 989 cm^−1^ and 1825 cm^−1^, respectively. This reduction indicates a nucleophilic attack by the oxygen atoms in FEC on Al(EtO)_3_, forming more stable Al–O bonds. Consequently, the original C=O bonds may cleave or undergo structural transformations, decreasing their absorption peak intensities. Further polymerization of FEC molecules likely releases HF (Fig. 2b) [28]. As shown in Fig. 2c, the FTIR analysis of PFPN reveals absorption peaks at 784 cm⁻^1^, 839 cm⁻^1^ and 1248 cm⁻^1^ corresponding to the P–N, P–F and P=N bonds, respectively. Additionally, absorption peaks for the P–O–C bond were observed at 938 cm⁻^1^ and 1012 cm⁻^1^ [24]. In the Al(EtO)_3_/PFPN sample, the intensities of the P–N, P=N and P–F absorption peaks significantly decreased. This change in absorption intensity can be attributed to the nucleophilic attack of the oxygen atom (–OEt) in Al(EtO)_3_ on the phosphorus in PFPN via an S_n2_ mechanism, reducing the electron density of the P–F bond in PFPN and making it more susceptible to attack, leading to substitution and initiating ring-opening reactions [29]. Meanwhile, Al^3+^ in Al(EtO)_3_ acts as a Lewis acid catalyst to stabilize the free fluoride ion [30]. Additionally, the intensity of the P–O–C absorption peak in PFPN markedly decreased, suggesting that the oxygen atom in PFPN might coordinate with aluminum, reducing the electron density and bond order of the P–O–C bond, as reflected in the weakened FTIR absorption peak. The formation of P–O–Al coordination disrupts the hydrogen bond network between Al(EtO)_3_ nanowires, decreasing inter-chain interactions and enhancing their dispersion in the electrolyte. Overall, the reaction between Al(EtO)_3_ and PFPN is driven by a synergy of nucleophilic substitution (EtO⁻ attack) and Lewis acid catalysis (Al^3+^ coordination), which together facilitate the ring-opening of PFPN and subsequent formation of cross-linked structures (Fig. 2d). Both FEC and PFPN share electron-deficient cyclic structures, in which the fluorine substituents (−F) on their rings exhibit strong electron-withdrawing effects. This characteristic renders them highly reactive toward nucleophilic agents, such as the ethoxy groups (−OEt) in Al(EtO)_3_, triggering ring-opening polymerization reactions. Additionally, both molecules incorporate electron-rich oxygen-containing groups (the carbonate group (C=O) in FEC and the ethoxy group (−OEt) in PFPN), which can engage in coordination interactions with the Al^3+^ center in Al(EtO)_3_, thereby forming a three-dimensional cross-linked network.

As shown in Fig. 2e, the carbonyl oxygen (C=O) in FEC donates its lone pair of electrons to the phosphorus atom in PFPN, forming a P–O coordination bond. This coordination reduces the electron density of the C=O bond in FEC, evidenced by the blue shift and decreased intensity of the C=O stretching vibration peak in the FTIR spectrum. The formation of the P–O coordination bond causes an electron redistribution within the PFPN ring, weakening the P–N bonds and destabilizing them. Consequently, the P–N single bonds in the PFPN ring are cleaved, leading to ring-opening and the generation of free nitrogen atoms. These free nitrogen atoms each form lone pairs that generate N–N single bonds, resulting in the stable dimerization of nitrogen (Fig. 2f). The FTIR spectra of PFPN/Al(EtO)_3_/FEC samples exhibit changes consistent with those observed in Al(EtO)_3_/PFPN, Al(EtO)_3_/FEC and PFPN/FEC samples, indicating that simultaneous reactions likely occur within the PFPN/Al(EtO)_3_/FEC mixture (Fig. 2g). To quantitatively assess the reaction kinetics, FTIR spectroscopy was employed to monitor the concentration variation of FEC. Given that FEC reacts with both PFPN and Al(EtO)_3_ at the C=O site, the change in the C=O peak intensity over time was used as an indicator of FEC concentration. Initially, standard FEC solutions (1–5 M) were prepared in diethylene glycol dimethyl ether, and the corresponding C=O peak intensities were measured to establish a calibration curve (Fig. S5a). Subsequently, the three additives were introduced proportionally into the solvent under simulated reaction conditions, and the concentration change of FEC was monitored over a 1-hour period. As depicted in Fig. S5b, the FEC concentration exhibited an approximately linear decline over 75 minutes, corresponding to a rate of change of about −0.000 128 M min^−1^. Thus, the reaction rate is estimated to be ∼0.000 128 M min^−1^.

Figure 2h presents the solid-state ^27^Al NMR spectrum of Al(EtO)_3_, revealing resonance peaks at 68, 36, 6 and –22 ppm, corresponding to various coordination environments of aluminum. The peaks at 68 ppm and 36 ppm represent four-coordinated (Al^Ⅳ^) and five-coordinated aluminum (Al^Ⅴ^), respectively, while the peaks at 6 ppm and –22 ppm correspond to six-coordinated aluminum (Al^Ⅵ^), confirming the structural characteristics of polymerized Al(EtO)_3_ nanowires [31,32]. In the Al(EtO)_3_/FEC samples, all Al^Ⅳ^ and Al^Ⅴ^ coordination states are converted to Al^Ⅵ^, consistent with our FTIR results, indicating effective coordination between FEC and Al(EtO)_3_. In the Al(EtO)_3_/PFPN mixture, compared to pure Al(EtO)_3_, the relative intensity of the Al^Ⅴ^ peak slightly decreases, which aligns with our FTIR analysis. When PFPN molecules coordinate with the oxygen atoms present in Al(EtO)_3_, they do not alter the coordination number of aluminum; however, when PFPN molecules coordinate with the aluminum atom of Al(EtO)_3_ through their own oxygen atoms, additional coordination bonds are formed, thereby promoting the conversion of aluminum from Al^Ⅴ^ to Al^Ⅵ^. During the nucleophilic attack of oxygen atoms in Al(EtO)_3_ on PFPN, the lone pair of oxygen electrons are transferred to the phosphorus atoms, reducing the electron cloud density around the oxygen atoms and diminishing their ability to donate electrons to aluminum. Additionally, the decreased electron density reduces the electronegativity of the oxygen atoms, resulting in reduced electron withdrawal from the aluminum atoms. Consequently, the electron cloud density around aluminum increases, enhancing the electron shielding effect and causing the chemical shift of aluminum to move towards higher field (lower ppm) regions. To investigate the effect of Al(EtO)_3_ on the solvation structure of the electrolyte, we characterized the solvation structure of the electrolyte using Raman spectroscopy. Figure S6 presents the Raman spectra of LiPF_6_, EC/DEC, BE and AE. In the Raman spectrum of EC/DEC, the peaks at 893 and 715 cm^−1^ correspond to the ring-breathing vibrations of EC. After the addition of LiPF_6_, new peaks appear at 902 and 740 cm^−1^, corresponding to the stretching vibration modes (v_3_ and v_1_) of PF_6_^−^, indicating the interaction between LiPF_6_ and the solvent [33]. With the further addition of Al(EtO)_3_, the positions of these peaks remain unchanged, suggesting that Al(EtO)_3_ has little effect on the solvation structure of the electrolyte. This is because the solubility of Al(EtO)_3_ in the electrolyte is far lower than that of LiPF_6_, and the coordinating ability of the EC/DEC mixed solvent is already fully occupied by Li^+^, forming a stable solvation shell; the trace addition of Al(EtO)_3_ is insufficient to disturb this balance. This result further supports our view that the primary function of PAFE is to improve the electrode/electrolyte interface (SEI/CEI layers), rather than exerting its effect by altering the solvation structure of the electrolyte.

Figure 2i illustrates the possible formation of highly complex polymers resulting from the interactions among Al(EtO)_3_, PFPN and FEC. In addition, GPC analysis revealed two main molecular weight ranges of ∼700 and 1600 (Fig. S7). This indicates that, besides pairwise reactions yielding lower-molecular-weight products (∼700), a synergistic reaction among all three additives produces higher-molecular-weight polymers (∼1600). This finding further supports our hypothesis that interactions among Al(EtO)_3_, PFPN and FEC generate complex polymer structures, which are crucial for achieving a uniform dispersion of inorganic components in the SEI/CEI layers. Consequently, this facilitates uniform ion transport and enhanced stability of the interfacial layers, ultimately improving the overall performance of the battery.

### *Ex situ* physicochemical characterizations of SEI layers and lithium deposition behavior in Li||Cu cells

**Figure 3.**
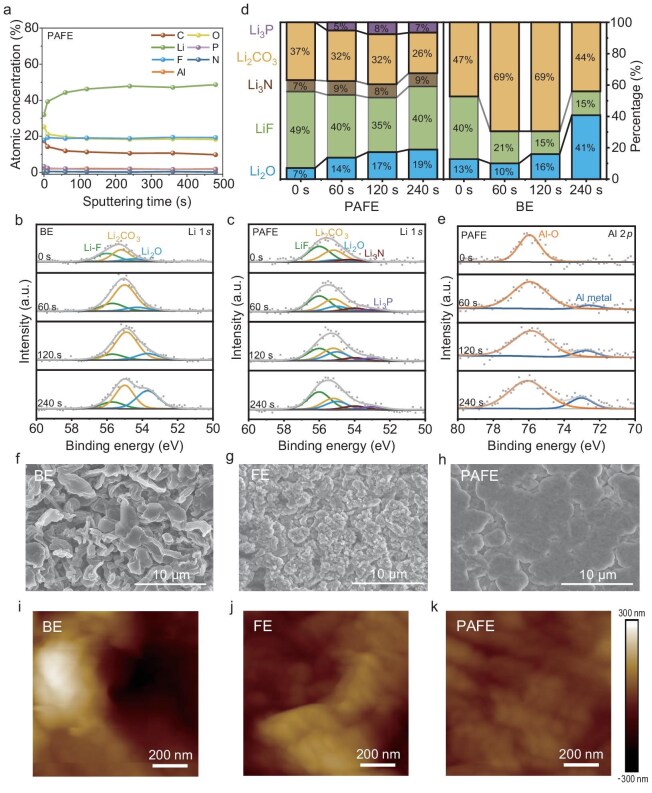
*Ex situ* physicochemical characterizations of SEI layers and lithium deposition behavior in Li||Cu cells. (a) Atomic concentration of detected elements on SEI formed in PAFE. XPS depth-profiles of Li 1 *s* spectra of LMAs after 10 cycles at 1 mA cm^−2^ and 1 mAh cm^−2^ in Li||Li coin cells with BE (b) and PAFE (c). (d) The relative composition of Li-containing species. (e) XPS Al 2*p* spectra. SEM images (f–h) and height maps (i–k) of the deposited Li metal using BE (f, i), FE (g, j) and PAFE (h, k) at 0.5 mA cm^−2^ and 3 mAh cm^−2^.

We utilized X-ray photoelectron spectroscopy (XPS) to analyze the chemical composition of the SEI on post-cycled LMAs. Cells were cycled for 10 cycles at 1 mA cm^−2^ and 1 mAh cm^−2^, and the cell voltage was reset to 0 V prior to disassembly. The F 1 *s* XPS spectra showed peaks at 684.8, 686 and 687.6 eV, corresponding to LiF, PO_x_F_y_ and C–F bonds, respectively (Fig. S8) [34,35]. In the BE, LiF and PO_x_F_y_ species arose from reactions of PF_5_/PF_6_^−^ groups with metallic lithium, leading to an SEI with limited LiF content and uneven distribution [36]. By contrast, PAFE exhibited a higher LiF content owing to the extra fluorine supplied by FEC and PFPN. LiF is known for its high mechanical strength and chemical stability, which enhances the SEI's ability to suppress lithium dendrite growth [16]. With prolonged Ar^+^ sputtering, the LiF content in BE decreased, indicating that LiF was primarily located in the outer layers of the SEI and was not uniformly distributed. Conversely, in PAFE, the LiF content remained stable across various depths. As illustrated in Fig. 3a, the fluorine fraction in PAFE consistently remained at ∼20% throughout etching, indicating a fluoride-rich and uniformly distributed SEI. However, the F content in BE gradually decreased (Fig. S9). This uniform distribution of LiF throughout the SEI contributes to consistent Li^+^ flux and enhanced interfacial stability.

The O 1 *s* spectra showed peaks at 527.5 eV (Li_2_O) and 531 eV(Li_2_CO_3_) [37] (Fig. S10). The PAFE electrolyte exhibited a lower Li_2_CO_3_ content compared to BE, consistent with C 1 *s* spectra findings (Fig. S11). This suggests that, in PAFE, lithium metal primarily reacts with the additives rather than the electrolyte solvents during SEI formation, resulting in an SEI rich in inorganic species rather than organic decomposition products. Additionally, the consistent concentrations of Li_2_CO_3_ and Li_2_O in PAFE over etching time indicate their uniform distribution within the SEI, further supporting the formation of a stable and homogeneous interfacial layer. The Li 1 *s* spectra of BE identified three components at ∼56 eV, 55.2 eV and 55 eV, assigned to LiF, Li_2_CO_3_ and Li_2_O (Fig. 3b) [37]. In PAFE, two new peaks appeared at ∼53.5 eV and 53 eV, attributed to Li_3_P and Li_3_N, decomposition products of PFPN (Fig. 3c) [38–40]. Compared to BE, these findings are corroborated by peaks in the P 2*p* and N 1 *s* spectra (Figs S12 and S13), confirming the successful incorporation of phosphorus and nitrogen species into the SEI. Li_3_N and Li_3_P are known for their high Li^+^ conductivity, which significantly enhances Li^+^ transport at the interface. Figure 3d presents the relative quantities of lithium-containing species at various depths based on the Li 1 *s* spectra results. The data indicate a predominant presence of LiF in the SEI, with PAFE exhibiting a richer and more uniformly distributed inorganic component compared to BE. This uniform distribution is essential for consistent ionic transport and mechanical stability across the SEI layer. Additionally, a peak at 74.9 eV in the Al 2*p* spectra indicates the presence of Al–O species (Fig. 3e), indicating the formation of Al_2_O_3_ within the SEI [28]. The Al–O content decreases with prolonged Ar^+^ etching, while metallic Al content rises, implying an Al-rich inner SEI layer. The nearly constant Al atomic fraction (∼2.2%) throughout etching confirms a uniform Al distribution in the SEI, contributing to the overall structural integrity and thermal stability of the interfacial layer.

XPS analysis reveals that the SEI generated in PAFE is rich in inorganic constituents such as Al_2_O_3_, LiF, Li_2_O, Li_3_N, Li_2_CO_3_ and Li_3_P, uniformly distributed throughout the SEI. The presence of these inorganic components enhances the SEI's mechanical strength, chemical stability, and ionic conductivity. Notably, Li_3_N and Li_3_P, both possessing high Li^+^ conductivity, markedly expedite interfacial ion transport. Concurrently, Al_2_O_3_, known for its chemical and thermal stability, contributes to a stable interfacial layer that resists degradation during cycling. The uniform distribution of these inorganic components within the SEI, combined with increased Li^+^ diffusion rates facilitated by high-conductivity species, ensures homogeneous Li^+^ flow. Such homogeneous Li^+^ flux minimizes local current hotspots, suppresses dendritic growth, and thereby improves cycling stability. Thus, the synergistic effects of PFPN, FEC and Al(EtO)_3_ within the electrolyte create a stable, inorganic-rich, and uniformly distributed SEI layer on the LMAs. This advancement not only improves cycling stability and Li^+^ transmission rates but also ensures uniform Li^+^ flow, thereby enhancing battery performance and safety.

A compositionally uniform SEI is expected to facilitate homogeneous ion flux and reduce local stress. Accordingly, we further verified the effectiveness of PAFE in suppressing dendrite formation and enhancing interfacial stability by conducting morphological and mechanical characterizations. We used scanning electron microscopy (SEM) to examine lithium deposition in various electrolytes after operating Li||Cu cells at 0.5 mA cm^−2^ for 6 hours with an areal capacity of 3 mAh cm^−2^. In BE, dendritic lithium growth was observed on the Cu electrode (Fig. 3f). This dendritic structure increases the reactive surface area, accelerates electrolyte decomposition, and leads to ‘dead-lithium’ formation, adversely affecting cycling performance. The uneven and fragile SEI in BE likely promotes non-uniform Li^+^ flux, fostering dendrite growth. Even with the addition of 20 vol.% FEC, lithium deposition remained heterogeneous and porous (Fig. 3g). Although FEC is known to form LiF-rich SEI layers that can improve anode stability, the SEI formed may not be sufficiently uniform or robust under these conditions to prevent lithium dendrite formation. This suggests that while FEC contributes to SEI formation, it alone is insufficient to achieve the desired uniformity and mechanical integrity to guide homogeneous lithium deposition. In contrast, incorporating PAFE resulted in a significantly flatter and denser lithium morphology (Fig. 3h). Although PAFE exhibits a slightly lower ionic conductivity (7.02 mS cm^−1^) and marginally higher viscosity (17.3 mPa s) at 25°C than BE (8.16 mS cm^−1^ and 16.3 mPa s, respectively), this can be attributed to its unique three-dimensional polymer network, which more effectively incorporates inorganic components (e.g. LiF, Li_3_N, Li_3_P and Al_2_O_3_) (Tables S2 and S3). These components enhance the mechanical robustness of the SEI/CEI, favoring uniform Li⁺ flux and ultimately suppressing dendritic lithium growth.

Deformation of the SEI during lithiation and delithiation is unavoidable, making a uniform and mechanically strong SEI essential for homogeneous lithium deposition and dendrite suppression. Atomic force microscopy (AFM) measurements revealed a more uniform SEI with PAFE (Fig. 3i–k), characterized by a significantly lower arithmetic mean roughness (R_a_) of 27.5 nm compared to 62.4 nm for BE and 49.8 nm for FE (Fig. S14). The lower roughness indicates a smoother and more homogeneous SEI surface, which promotes even Li^+^ distribution during deposition. Additionally, force–displacement curves (Fig. S15) show the highest adhesion force for the PAFE-formed interphase, indicating a denser and more cohesive SEI layer with superior mechanical properties. This enhancement is attributed to the uniform incorporation of inorganic components like Al_2_O_3_ within the SEI, improving its adhesion and resistance to cracking or delamination. Contact-angle tests confirmed that PAFE exhibits better wettability on polypropylene separators (Fig. S16), promoting micro-homogeneity during SEI formation. Improved wettability ensures more intimate contact between the electrolyte and the electrode surface, thereby facilitating the uniform deposition of SEI components. This leads to a consistent SEI layer that effectively regulates Li^+^ flux and suppresses lithium dendrite formation. In addition, observations of the LMAs thickness evolution during long-term cycling (Fig. S17) further corroborate this conclusion. Although the Li metal undergoes some degree of volumetric expansion in both BE and PAFE electrolytes, the expansion is notably smaller in the PAFE system. This finding suggests that the various components in PAFE interact to form a three-dimensional polymer network, effectively buffering the LMAs against volume changes during repeated lithiation and delithiation cycles.

Overall, the compact, mechanically robust SEI formed in the PAFE electrolyte facilitates uniform Li^+^ deposition, suppresses dendrite formation, and thus enhances ion transport, battery performance and safety. These results accord with our earlier discussions on the synergistic effects of Al(EtO)_3_, PFPN and FEC in forming homogeneous interfacial layers rich in inorganic components. The unique composition and uniform distribution of SEI components in PAFE play a critical role in improving the morphology of lithium deposition and, consequently, the overall performance of the battery.

### Electrochemical measurements and analysis of Li||Cu and Li||Li coin cells

**Figure 4.**
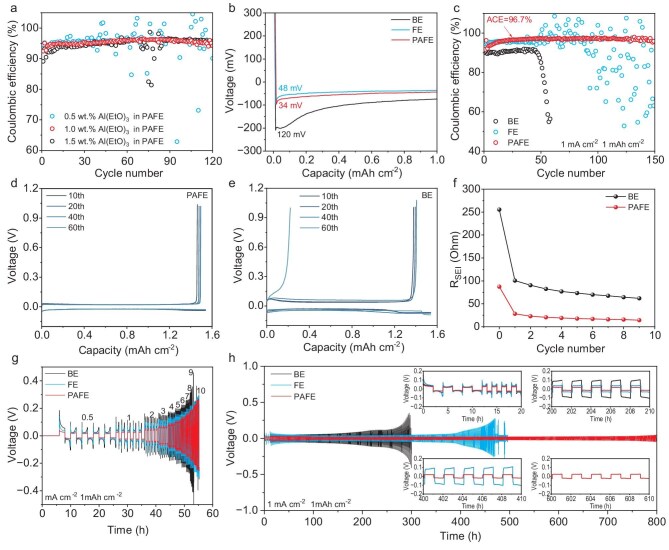
Electrochemical measurements and analysis of Li||Cu and Li||Li coin cells. (a) Coulombic efficiency of the Li||Cu cells with different Al(EtO)_3_ contents. Overpotential during the first lithium plating cycle (b), Coulombic efficiency (c) and voltage profiles (d, e) of the Li||Cu cells. (f) *R*_SEI_ of the Li||Li symmetric cells with increasing cycle numbers. Rate capability (g) and voltage–time profiles (h) of Li||Li symmetric cells with different electrolytes. Insets are the enlarged figures of the curves.

To explore the appropriate amount of Al(EtO)_3_ nanowire additive, we tested and compared the Coulombic efficiency of Li||Cu cells using PAFE electrolytes containing 0.5, 1.0 and 1.5 wt.% Al(EtO)_3_. As shown in Fig. 4a, 1.0 wt.% Al(EtO)_3_ is optimal for achieving a robust and uniform SEI, as lower concentrations (0.5 wt.%) do not sufficiently promote the formation of the three-dimensional network, while higher concentrations (1.5 wt.%) lead to incomplete dissolution and inferior dispersion. To further evaluate the effectiveness of the SEI in enhancing the reversibility of lithium deposition, we assembled Li||Cu cells using BE, FE and PAFE electrolytes. All cells were discharged to a fixed capacity of 1 mAh cm^−2^ and then charged to 1 V at a current density of 1 mA cm^−2^ to strip lithium from the copper foil. In the initial discharge cycle of the Li||Cu cells, the SEI layer formed in PAFE facilitated rapid and uniform Li^+^ transport, resulting in the lowest deposition overpotential among the tested electrolytes (Fig. 4b). This improved ionic conduction significantly contributed to spherical lithium deposition on the Cu substrate, effectively mitigating lithium dendrite formation. As depicted in Fig. 4c, cells using BE started with an initial Coulombic efficiency of 89%, which precipitously declined to below 50% after 70 cycles. This decline can be attributed to the uneven SEI layer formed in BE, leading to increased side reactions and the formation of ‘dead-lithium’ thereby reducing the reversibility of lithium plating and stripping. In comparison, cells with FE initially reached a Coulombic efficiency of 95% within 50 cycles, but then experienced notable efficiency fluctuations in subsequent cycles, suggesting insufficient SEI stability over prolonged cycling. In stark contrast, PAFE-based cells consistently achieved an average Coulombic efficiency of 96.7% across 150 cycles. This high and stable Coulombic efficiency is attributed to the synergistic effects of Al(EtO)_3_, PFPN and FEC in forming a uniform, inorganic-rich SEI layer. The presence of uniformly distributed inorganic components such as LiF, Li_3_N, Li_3_P and Al_2_O_3_ enhances the SEI's mechanical strength and ionic conductivity, effectively suppressing side reactions and lithium dendrite growth. Consequently, this leads to highly reversible lithium deposition and dissolution processes. Voltage profiles presented in Fig. 4d, e and Fig. S18 indicated that PAFE cells exhibited stable polarization during lithium plating and stripping processes, without the abrupt increases observed in cells formulated with BE and FE. The stable voltage profiles further confirm the formation of a robust SEI layer in PAFE that maintains consistent ionic transport and minimizes resistance fluctuations during cycling.

To further elucidate the stability and ion transport properties of the SEI layer, EIS measurements were conducted on Li||Li symmetric cells. The cells underwent charge and discharge cycles at a current density of 1 mA cm^−2^ and a fixed deposition capacity of 1 mAh cm^−2^, with EIS spectra recorded after each cycle (Fig. S19). In the high-frequency region of the Nyquist plot, the semicircle represents the migration of Li^+^ through the SEI. For non-cycled cells, the *R*_SEI_ in BE reached a high value of 220.7 Ohms, indicating an initially uneven and less dense SEI that hinders Li^+^ transport. In contrast, the non-cycled *R*_SEI_ in PAFE was significantly lower at 84.09 Ohms, suggesting that the initial SEI formed in PAFE is more conducive to ion transport due to its uniform composition and structure. After four cycles, the *R*_SEI_ in PAFE decreased to 17.34 Ohms, only 20% of its initial value, and continued to show a decreasing trend before stabilizing in subsequent cycles (Fig. 4f). This reduction indicates that the SEI in PAFE becomes more stable and facilitates easier Li^+^ migration as cycling progresses. In comparison, *R*_SEI_ values for BE exhibited considerable fluctuations and remained ∼400% higher than those in PAFE at the same cycle number. The high and unstable *R*_SEI_ in BE implies continuous SEI formation and degradation during cycling, which hampers Li^+^ transport and affects cell performance. These observations suggest that the reaction products of Al(EtO)_3_, PFPN and FEC in PAFE contributed to the uniform formation of a stable SEI during cell standstill and early cycling stages. The macromolecular complexes formed through their interactions contribute to a stable and uniformly distributed inorganic content within the SEI. This optimized SEI structure facilitates the unimpeded movement of Li^+^ across the SEI, thereby lowering the activation energy and enhancing ionic conductivity.

In terms of rate performance and cycling stability in Li||Li symmetric cells, PAFE exhibits superior properties compared to BE and FE. As demonstrated in Fig. 4g, at an areal capacity of 1 mAh cm^−2^, the current density incrementally increased from 0.5 to 10 mA cm^−2^. In the initial cycle, PAFE displayed very low nucleation overpotential and polarization voltage, indicating efficient lithium deposition kinetics. As the current density escalated, both BE and FE showed increased polarization voltages relative to PAFE, reflecting higher resistance and less efficient ion transport. When the current density reached 9 mA cm^−2^, the BE cell failed due to its inability to withstand the increased stress, likely caused by uneven lithium deposition and dendrite formation. In contrast, the PAFE cell maintained stable operation even at high current densities, underscoring the robustness of the SEI layer formed in PAFE. In Fig. 4h, at a current density of 1 mA cm^−2^ and an areal capacity of 1 mAh cm^−2^, PAFE exhibited stable cycling for >800 hours, with a smaller and more consistent polarization voltage. This long-term stability demonstrates the effectiveness of the uniform, inorganic-rich SEI in suppressing lithium dendrite growth and maintaining efficient lithium plating and stripping over extended cycles. In contrast, BE and FE cells quickly short-circuited after an increase in polarization voltages, indicative of dendrite-induced failures due to unstable SEI layers.

Overall, the enhanced electrochemical performance observed with PAFE can be attributed to the formation of a stable, inorganic-rich SEI layer that promotes uniform Li^+^ flow and suppresses lithium dendrite growth. The synergistic effects of Al(EtO)_3_, PFPN and FEC not only enhance the ionic conductivity and mechanical strength of the SEI but also significantly reduce the E_a_ for Li^+^ migration. This comprehensive improvement facilitates highly reversible lithium plating and stripping, thereby enhancing battery performance and safety.

### Electrochemical characterization of the Li||NCM811 cells

**Figure 5.**
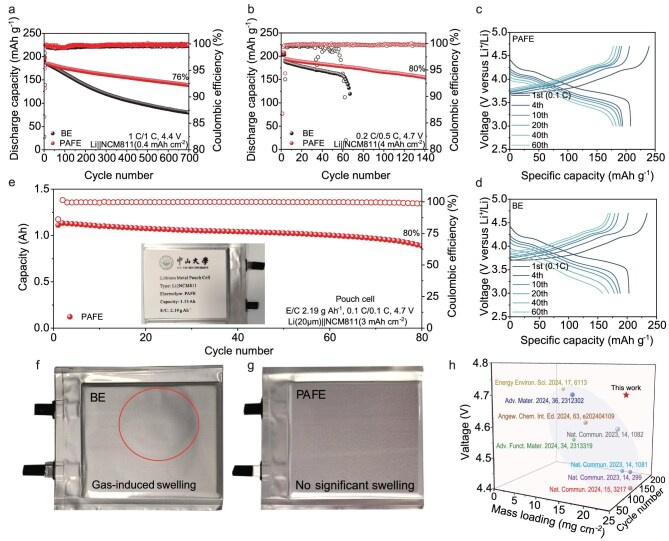
Electrochemical characterization of the Li||NCM811 cells. (a) Cycling performance of the coin cells at 1 C charge/1 C discharge in the voltage range of 3.0–4.4 V. (b) Cycling performances of the coin cells at 0.2 C charge/0.5 C discharge in the voltage range of 3.0–4.7 V. Corresponding selected charge/discharge curves in PAFE (c) and BE (d). (e) Cycling performance of the 1 Ah Li||NCM811 pouch cell operated at 0.1 C charge/0.1 C discharge in the voltage range of 3.0–4.7 V. Volumetric changes in 1 Ah lithium metal pouch cells using BE (f) and PAFE (g) electrolytes during cycling. The BE-based cell showed significant swelling after 20 cycles at 4.3 V, while the PAFE-based cell exhibited no noticeable expansion after 80 cycles at 4.7 V, demonstrating the effective suppression of gas generation by PAFE. (h) Outperforming recent state-of-the-art electrolytes.

To evaluate the cyclic stability of PAFE in LMBs under high-voltage conditions, we assembled full cells using NCM811 as cathodes and lithium metal foils as anodes. These cells were initially activated at a current density of 0.1 C (1 C = 200 mA g^−1^) before undergoing extended cycling tests. In experiments with Li||NCM811 coin cells featuring a cathode loading of 2 mg cm^−2^ and cycled at 1 C with a cut-off voltage of 4.4 V, cells with BE degraded rapidly after 100 cycles due to accelerated lithium dendrite formation and electrolyte decomposition. In contrast, cells with PAFE retained 75.8% of their capacity after 700 cycles, exhibiting significantly reduced polarization (Fig. 5a and Fig. S20). This enhanced performance is attributed to the robust and uniform SEI and CEI layers formed by PAFE, which suppress lithium dendrite growth and stabilize the electrolyte under high-voltage conditions. Further tests on Li||NCM811 coin cells with a higher cathode loading of 21.5 mg cm^−2^, cycled at 0.2 C for charging and 0.5 C for discharging at a cut-off voltage of 4.4 V, revealed that PAFE maintained 78% capacity after 180 cycles, significantly outperforming BE with much lower polarization (Fig. S21). This indicates that PAFE effectively mitigates issues associated with high mass loading, such as increased internal resistance and electrolyte degradation, by forming stable interfacial layers that facilitate efficient ion transport.

High-energy-density batteries are pivotal for advancing consumer electronics, electric transportation and renewable-energy storage solutions. Achieving such high energy densities requires elevating the battery cut-off voltage, which imposes stringent performance criteria on the electrolyte due to increased oxidative stress and cathode instability. Utilizing Li||NCM811 coin cells with cathode mass loadings of 21.5 mg cm^−2^, operated at 0.2 C for charging and 0.5 C for discharging at a cut-off voltage of 4.7 V, we observed that BE cells experienced rapid capacity decay after 60 cycles. In contrast, PAFE cells retained 80% capacity after 140 cycles (Fig. 5b), demonstrating exceptional stability under high-voltage conditions and outperforming recent state-of-the-art electrolytes (Fig. 5h and Table S4). PAFE-based cells also exhibited significantly lower polarization across varying cycles (Fig. 5c, d). Beyond its outstanding cycling stability, the self-discharge behavior of the PAFE electrolyte was also evaluated. Li||NCM811 cells using the PAFE electrolyte were charged to 4.7 V and then rested for 1 week. As shown in Fig. S22, the voltage decay for the PAFE electrolyte was significantly lower (0.2359 V) compared to that of BE (0.2468 V), indicating a reduced self-discharge rate. This result further underscores the stability of the interfacial layers formed by PAFE, contributing to the overall superior performance of the battery. Even at an elevated cut-off voltage of 4.9 V, Li||NCM811 cells with PAFE cycled steadily for 70 cycles (Fig. S23), underscoring the electrolyte's superior oxidative stability and interfacial compatibility. To evaluate the temperature tolerance of Li||NCM811 cells with PAFE electrolyte, we tested their cycling performance under high and low temperature conditions. As shown in Fig. S24a, at 60°C the PAFE‑based cell retains far more capacity over prolonged cycling than its BE counterpart; similarly, at 0°C (Fig. S24b) PAFE again outperforms BE by a wide margin. This superior high‑/low‑temperature performance is attributed to the robust, uniform SEI/CEI and three‑dimensional polymer network formed in PAFE, which both stabilizes the electrode interface at elevated temperatures and promotes efficient ion transport at subzero temperatures. Additionally, we have tested the cycling performance of Li||NCM811 cells with FE and PE. The results show that although FE and PE improve capacity retention versus BE, their performance remains inferior to that of the PAFE system (Fig. S25). This is attributed to the fact that the synergistic reaction among Al(EtO)_3_ nanowires, PFPN and FEC in PAFE is essential for establishing a uniform and low-resistance interphase.

Recent studies on high-voltage batteries often employ low-loading cathodes or excess electrolytes, which do not accurately reflect conditions in practical batteries. In our study, we utilized a high-loading cathode (2.85 mAh cm^−2^), low N/P ratio (1.44), and limited electrolyte (2.19 g Ah^−1^) to test 1 Ah pouch cells at 4.7 V (Table S5). The 4.7 V rechargeable Li||NCM811 pouch cell cycled in PAFE retained 80% capacity after 80 cycles and showed very low polarization across varying cycles (Fig. 5e and Fig. S26). Post-cycling comparisons of pouch cells revealed that the 1 Ah lithium metal pouch cell using BE swelled markedly after 20 cycles at 4.3 V (Fig. 5f), whereas the PAFE cell showed no discernible expansion after 80 cycles at 4.7 V (Fig. 5g). This indicates that PAFE effectively suppresses the generation of gas during cycling. These results demonstrate PAFE's ability to maintain high performance under stringent conditions that closely mimic commercial battery requirements. In addition to the above performance tests, we further evaluated the shelf-life stability of the PAFE electrolyte. Li||NCM811 cells were assembled using freshly prepared PAFE electrolyte as well as electrolyte stored for 1 month. As shown in Fig. S27, both sets of cells exhibited nearly identical cycling performance, indicating that the PAFE retained its electrochemical properties after 1-month storage.

### *Ex situ* post-mortem physicochemical characterizations of the NCM811 cathodes

**Figure 6.**
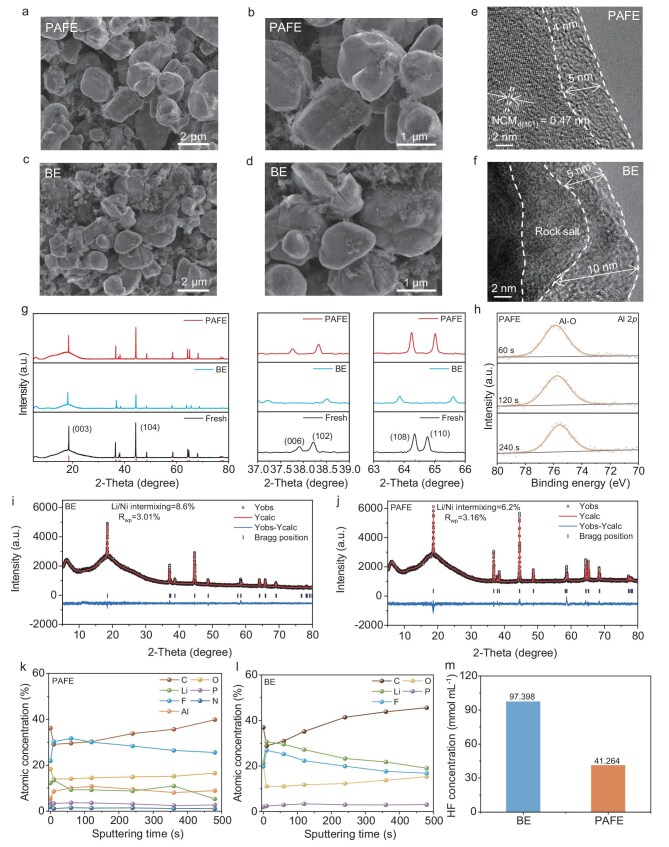
*Ex situ* post-mortem physicochemical characterizations of the NCM811 cathodes. SEM images of the NCM811 cathode cross-section after 50 cycles in PAFE (a, b) and BE (c, d). TEM analyses of the NCM811 particles after 50 cycles in PAFE (e) and BE (f). (g) XRD of the NCM811 cathodes before and after 50 cycles in PAFE and BE. (h) XPS spectra of the Al 2*p*. Rietveld refinement on the XRD data of samples cycled with BE (i) and PAFE (j). Atomic ratio of elements of the NCM811 cathodes in PAFE (k) and BE (l). (m) HF concentration in BE + 1000 ppm H_2_O and PAFE + 1000 ppm H_2_O.

To elucidate the mechanism behind the stable cycling performance of NCM811 full cells utilizing PAFE, we characterized high-loading NCM811 cathodes after 50 cycles in different electrolytes (loading: 21.5 mg cm^−2^, charge/discharge current density: 0.3 C, voltage range: 3.0–4.5 V, disassembled at 3.0 V). SEM analyses revealed that electrodes cycled with PAFE exhibited intact NCM811 particles without noticeable cracks (Fig. 6a, b and Fig. S28), indicating that the CEI formed in PAFE facilitates rapid and homogeneous Li^+^ transport, thereby mitigating internal stress. In contrast, significant cracking and fragmentation were observed in the cross-sections and surfaces of cathode particles cycled with BE (Fig. 6c, d and Fig. S28). This degradation is attributed to uneven Li^+^ intercalation and deintercalation, leading to differential volume expansion and contraction, which induces material stress and subsequent crack formation. Further insights were obtained through transmission electron microscopy (TEM) characterization of the CEI on NCM811 cathodes (Fig. 6e, f). The CEI formed in BE was thick and uneven, and a rock-salt transition layer appeared near the surface, thereby diminishing the electrochemical activity of the material. In stark contrast, the CEI layer in PAFE was notably thinner (∼4 nm) without the formation of the rock-salt phase.

After 50 cycles, we conducted X-ray diffraction (XRD) analysis of the NCM811 cathode materials to verify the enhanced structural stability imparted by the PAFE electrolyte. The conventional XRD patterns (Fig. 6g) show that the NCM811 cathode cycled with PAFE retained a structure closer to that of the pristine material compared to the one cycled with BE. This was evidenced by a closer (003)/(104) peak intensity ratio and separation between the (006)/(102) and (108)/(110) peaks in the PAFE-cycled sample, suggesting better preservation of the layered structure [41,42]. To quantitatively assess the degree of cation mixing, we performed Rietveld refinement on the XRD data of samples cycled with BE and PAFE (Fig. 6i, j). The refinement results revealed that the Li/Ni cation mixing in the PAFE sample was 6.2%, far lower than the 8.6% in the BE sample. This reduction in cation mixing indicates that the PAFE electrolyte effectively suppresses the migration of Ni^2+^ ions into the Li layers, thereby preserving the integrity of the layered structure. To further elucidate the relationship between the uniformity of ion flux and stress evolution, we performed a lattice strain analysis based on XRD data and the lattice constants obtained via Rietveld refinement (Table S6). The c/a lattice constant ratio serves as a conventional indicator for evaluating lattice strain and anisotropic variations [43]. Compared to the fresh electrode (c/a ratio stable at 4.941), the PAFE system showed minimal variation in the c/a lattice constant ratio (from 4.941 to 4.978, Δ=0.76%), suggesting that uniform Li-ion flux suppresses anisotropic strain accumulation. In contrast, the BE system exhibited a larger variation (from 4.941 to 5.081, Δ=2.83%), indicating that non-uniform ion flow induces greater lattice strain and stress. This quantitative evidence directly supports the coupling between uniform ion transport and reduced stress evolution. Combining the conventional XRD data with the Rietveld refinement analysis, we conclude that the PAFE electrolyte maintains the NCM811’s layered structure by reducing cation mixing and strain stress, thereby significantly enhancing the cathode's structural stability under high-voltage cycling conditions. This enhanced structural stability is crucial for prolonging battery life and improving performance.

Further supporting these findings, XPS analysis (Fig. 6h, k, l) confirmed a uniform distribution of various inorganic components, including Al_2_O_3_, on the surfaces of NCM811 particles. The presence of these inorganic substances contributes to the formation of a robust CEI, enhancing structural stability by suppressing surface side reactions and reducing transition metal dissolution (detailed C 1*s*, O 1*s* and F 1*s* XPS spectra of the CEI are provided in Figs S29–31). These results demonstrate that the PAFE electrolyte not only improves the electrochemical performance but also enhances the structural integrity of high-voltage NCM811 cathodes through the suppression of cation mixing and stabilization of the layered structure. The ability of PAFE to maintain the cathode's structural integrity aligns with our design strategy of forming a uniform interphase that facilitates homogeneous Li^+^ transport and reduces internal stress within the electrode materials.

Moreover, PAFE can reduce the HF content in the electrolyte, primarily due to the presence of Al(EtO)_3_, which effectively removes trace amounts of water. The HF in the electrolyte mainly originates from the hydrolysis of LiPF_6_, a process involving two steps (Reactions 1 and 2) [44], with Reaction 1 being the rate-determining step. Since the hydrolysis of LiPF_6_ itself is inherently slow [45], Al(EtO)_3_, as a highly reactive metal alkoxide, rapidly consumes trace water in the electrolyte through a vigorous reaction (Reaction 3) [46]. This process effectively suppresses the occurrence of Reactions 1 and 2, thereby reducing HF generation. Additionally, the generated Al_2_O_3_ also has the effect of removing the already produced HF [47].


(1) \begin{eqnarray*}
{\rm LiP{F}_6} + {\rm {H}_2O} \to {\rm LiF} + {\rm P{F}_5} + {\rm {H}_2O}
\end{eqnarray*}

(2) \begin{eqnarray*}
{\rm P{F}_5} + {\rm {H}_2O} \to {\rm PO{F}_3} + {\rm 2HF}
\end{eqnarray*}

(3) \begin{eqnarray*}
{\rm 2Al{({EtO})}_3} + {\rm 3{H}_2O} \to {\rm A{l}_2{O}_3} + {\rm 6 EtOH}
\end{eqnarray*}

To evaluate the effectiveness of PAFE in reducing HF content, we added 1000 ppm of H_2_O to the prepared BE and PAFE electrolytes and measured the HF content using an automatic electrochemical titrator. The results showed that PAFE significantly reduced the HF concentration compared to BE (Fig. 6m). By minimizing HF generation in the electrolyte, PAFE preserves the structural integrity and interfacial stability of the cathode material, thereby markedly enhancing battery performance, cycle life and safety.

## CONCLUSION

In this study, we developed a multifunctional electrolyte (PAFE) incorporating Al(EtO)_3_ nanowires, which effectively forms uniform and robust interphases on both LMAs and NCM cathodes. The homogeneous distribution of inorganic components within the SEI and CEI significantly reduces Li^+^ diffusion barriers and enables homogeneous ionic flux across the electrodes, thereby alleviating localized internal stress. The successful demonstration of PAFE in high-loading NCM811 cathodes and 1 Ah pouch cells underscores its commercial potential. This strategy paves the way for the design of electrolytes capable of stabilizing next-generation batteries. Future research should focus on scaling up the synthesis of Al(EtO)_3_ nanowires and exploring the compatibility of PAFE with other high-capacity cathode materials.

## METHODS

Detailed preparation and characterization methods for materials are available in the online Supplementary data.

## Supplementary Material

nwaf182_Supplemental_File
