# Supplementary material for: Facilitating uniform lithium-ion transport via polymer-assisted formation of unique interfaces to achieve a stable 4.7 V Li metal battery
Source: Natl Sci Rev. 2025 May 10;12(6):nwaf182. doi: 10.1093/nsr/nwaf182 (PMC12139002; doi:10.1093/nsr/nwaf182)
Supplement: nwaf182_Supplemental_File [file nwaf182_supplemental_file.pdf]

Supporting Information

Facilitating uniform lithium-ion transport via polymer-assisted formation of unique interfaces to achieve stable 4.7 V Li metal battery

Xinqi Li+, Zhaojie Li+, Chuang Li, Fei Tian, Zhengping Qiao, Danni Lei* and Chengxin Wang*

State Key Laboratory of Optoelectronic Materials and Technologies, School of Materials Science and Engineering, Sun Yat-Sen (Zhongshan) University, Guangzhou 510275, China

***Corresponding authors**. E-mails: leidanni@mail.sysu.edu.cn; wchengx@mail.sysu.edu.cn

+Equally contributed to this work.

Experimental Section

Materials: The commercial NCM811 electrode (with a mass loading of 21.5 mg cm^{-2}, 94.5 wt.% active material, and a thickness of 70 μm) was provided by Guangdong Canrd New Energy Technology Co., Ltd. The preparation of a 2 mg cm^{-2} mass loading NCM811 cathode was carried out as follows: A mixture of 80 wt.% NCM811 powder, 10 wt.% acetylene black, and 10 wt.% polyvinylidene fluoride (PVDF) was manually ground in an agate mortar for 30 minutes. The resulting mixture was stirred in N-methyl-2-pyrrolidone (NMP, Macklin) for 4 hours to form a slurry. The electrode slurry was then pasted onto aluminum foil (99.35%, 16 μm, HF-Kejing Co., Ltd., China) and dried in a vacuum oven at 80 °C for 12 hours. Ethylene carbonate (EC, 99.99%, battery grade) and diethyl carbonate (DEC, 99.99%, battery grade) were purchased from Duoduo Chemical Reagent Co., Ltd. The basic electrolyte BE (1 M $LiPF_6$ in EC and DEC at a 1:1 volume ratio, with H_2O < 10 ppm) and fluoroethylene carbonate (FEC, H_2O < 10 ppm) were purchased from Duoduo Chemical Reagent Co., Ltd. The pentafluorocyclotriphosphazene (PFPN, 99%, Macklin) was dried overnight with 4 Å molecular sieves (Sigma-Aldrich) and stored in an argon-filled glovebox (Mikrouna) (with H_2O < 0.01 ppm and O_2 < 0.01 ppm) before use. All chemicals were of analytical grade or higher and used without further purification unless stated otherwise.

Preparation of the $Al(EtO)_3$-containing functional electrolyte (PAFE): The production details of aluminum ethoxide ($Al(EtO)_3$) nanowires are as follows: A graphite crucible containing aluminum powder (0.3 g) and lithium particles (0.085 g) in a 1:1 atomic ratio was heated in a muffle furnace at 800 °C for 30 minutes. After natural cooling, a LiAl alloy plate was obtained. The alloy plate was polished and then reacted with ethanol at 60 °C for 30 hours, resulting in the formation of a gel containing $Al(EtO)_3$ nanowires. For the preparation of PAFE, 1 wt.% $Al(EtO)_3$ nanowires, 10 vol.% PFPN, and 20 vol.% FEC were added to BE. The mixture was subjected to ultrasonication at 35 °C for 2 hours before use.

Fabrication of the cells: The NCM811 cathode (diameter of 12 mm, thickness of 70 μm), paired with a commercial lithium metal anode (99.95%, 400 μm, provided by China Energy Lithium Co., Ltd.), along with a separator (Celgard 2500 porous polypropylene membrane), were assembled into CR2032 coin cells within an argon-filled glovebox. The electrolyte injection volume was 30 μL for Li||Li, Li||Cu (utilizing 9 μm Cu metal foil with 99.8% purity, sourced from Guangdong Canrd New Energy Technology Co., Ltd.), Li||NCM811 (with a loading of 2 mg cm^{-2}), and Li||NCM811 (with a loading of 21.5 mg cm^{-2}) coin cells. All cells were tested using freshly prepared electrolyte solutions.

Fabrication and testing of pouch cells: For practical application testing, 1.0 Ah flexible pouch cells were assembled using high-loading NCM811 cathodes (15.12 mg cm^{-2}) and lithium metal anodes (20 μm thick). The cathode sheets were prepared by coating the NCM811 slurry onto aluminum foil, followed by drying and calendaring to achieve the desired loading and thickness. The anode was a lithium metal foil laminated onto a copper current collector. A trilayer polypropylene (PP)

separator was used. The electrolyte amount was controlled at 2.19 g Ah^{-1}. The pouch cells were sealed under vacuum in aluminum-laminated film and tested using a battery testing system (LAND CT3002K, Land Electronic Co., Ltd., Wuhan). Cycling tests were performed at 0.1 C charge and 0.1 C discharge rates between 3.0 V and 4.7 V.

Physicochemical measurements: All sample preparation and handling were conducted in an argon-filled glovebox (H_2O < 0.1 ppm, O_2< 0.1 ppm) unless otherwise stated. Material morphology and composition were analyzed using scanning electron microscopy (SEM, Regulus 8230, Hitachi, Japan) and transmission electron microscopy (TEM, FEI Talos F200X, USA). For TEM, cycled electrodes were disassembled in an argon-filled glovebox. drop-casted onto carbon-coated grids, and dried under vacuum. X-ray photoelectron spectroscopy (XPS, ESCALab Xi+) was employed to analyze the surface chemical states of the lithium metal anode and NCM811 cathode. The etching times were set at 0, 60, 120 and 240 seconds for depth profiling. X-ray diffraction (XRD) was carried out using an Empyrean powder X-ray diffractometer with a Cu Kα radiation source. Fourier-transform infrared spectroscopy (FTIR) was performed using a Frontier FTIR instrument at a resolution of 0.5 cm^{-1}. Raman spectroscopy was performed using a Micro-Raman Polarization Imaging Spectrometer (Thermo Fisher DXR3xi). Solid-state nuclear magnetic resonance (NMR) spectra were acquired at 25 °C and 12 kHz on a Bruker AVANCE 600 superconducting NMR spectrometer. Surface mechanical properties of the solid–electrolyte interphase (SEI) were investigated via atomic force microscopy (AFM, Bruker Dimension Fastscan). Force–displacement curves were acquired with a silicon nitride cantilever (nominal spring constant 0.2 N m^{-1}), from which adhesion forces and elastic moduli were extracted. Wettability was evaluated by depositing ~5 μL of each electrolyte on a polypropylene separator and measuring contact angles (Krüss DSA100). Ionic conductivities (DDS-307A) and viscosities (Brookfield DV-III Ultra, 250 rpm, 60 s) were measured at 25 °C. Molecular weight distributions were determined by gel permeation chromatography (Waters 1515, THF as mobile phase). Flame resistance was assessed by placing 0.5 mL of each electrolyte in a ceramic dish and igniting with a butane torch to record ignition and total burning times. HF content was quantified by potentiometric titration (Metrohm 848 Titrino Plus) using a fluoride ion-selective electrode; samples with added 1000 ppm H_2O were similarly prepared to examine water-induced HF generation. After cycling, electrodes were rinsed with diethyl carbonate (DEC), vacuum-dried at 25 °C, and transferred in sealed holders to prevent contamination.

Electrochemical characterizations: To assess the cycling and rate performance of the manufactured coin cells, they were placed in a culture chamber to maintain a constant operating temperature of 30 ± 1 °C. Measurements were conducted using a battery testing system (LAND CT3002A, Land Electronic Co., Ltd., Wuhan). For Coulombic efficiency measurements, Li||Cu cells were cycled at a current density of 1 mA cm^{-2}. In each cycle, lithium was plated onto the copper electrode to a capacity of 1 mAh cm^{-2}, followed by stripping to 1.0 V. The Coulombic efficiency was calculated as the ratio of the stripping capacity to the plating capacity. The cells were

cycled continuously to evaluate the long-term efficiency. Li||Li symmetric cells were assembled using two lithium metal electrodes (diameter of 14 mm, thickness of 400 μm) separated by a Celgard 2500 membrane. The electrolyte volume was 30 μL. The cells were cycled at various current densities (from 0.5 mA cm^{-2} to 10 mA cm^{-2}) with a fixed capacity of 1 mAh cm^{-2} to evaluate the rate performance and long-term cycling stability. Electrochemical impedance spectroscopy (EIS) was performed at 30 °C using an electrochemical workstation (IVIUM Vertex) in potentiostatic mode over a frequency range from 100 kHz to 10 mHz with an amplitude of 5 mV, recording 10 data points per decade. The open-circuit voltage was applied for 30 seconds before carrying out the EIS measurements. Temperature-dependent EIS measurements were conducted on Li||Li symmetric cells to calculate the activation energy for lithium-ion diffusion through the SEI. The cells were cycled ten times at 1 mA cm^{-2} with a capacity of 1 mAh cm^{-2} to stabilize the SEI. EIS spectra were recorded at temperatures ranging from 10 °C to 35 °C, controlled by a climatic chamber (Binder MKF 240). The data were fitted using the Arrhenius equation to determine the activation energies. The activation energies for lithium-ion diffusion through the SEI and CEI were calculated using the Arrhenius equation: $\frac{T}{R} = Aexp\left(-\frac{E_a}{R_0 T}\right)$, where R is the resistance obtained from EIS fitting, A is the pre-exponential factor, Ea is the activation energy, R_0 is the gas constant, and T is the temperature in Kelvin. The natural logarithm of the resistance was plotted against the inverse of temperature (Arrhenius plot), and Ea was determined from the slope of the linear fit. Voltage profiles were recorded during galvanostatic cycling to analyze the overpotentials. The overpotentials were calculated by measuring the voltage difference between the plating and stripping processes at the same capacity.

Supplementary Figures

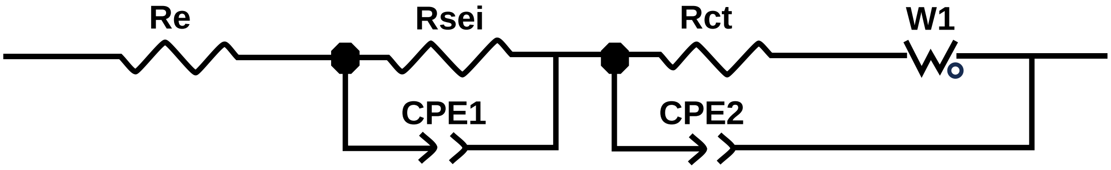

Figure S1. Equivalent circuit model used to fit the Electrochemical Impedance Spectroscopy (EIS) curves at different frequencies.

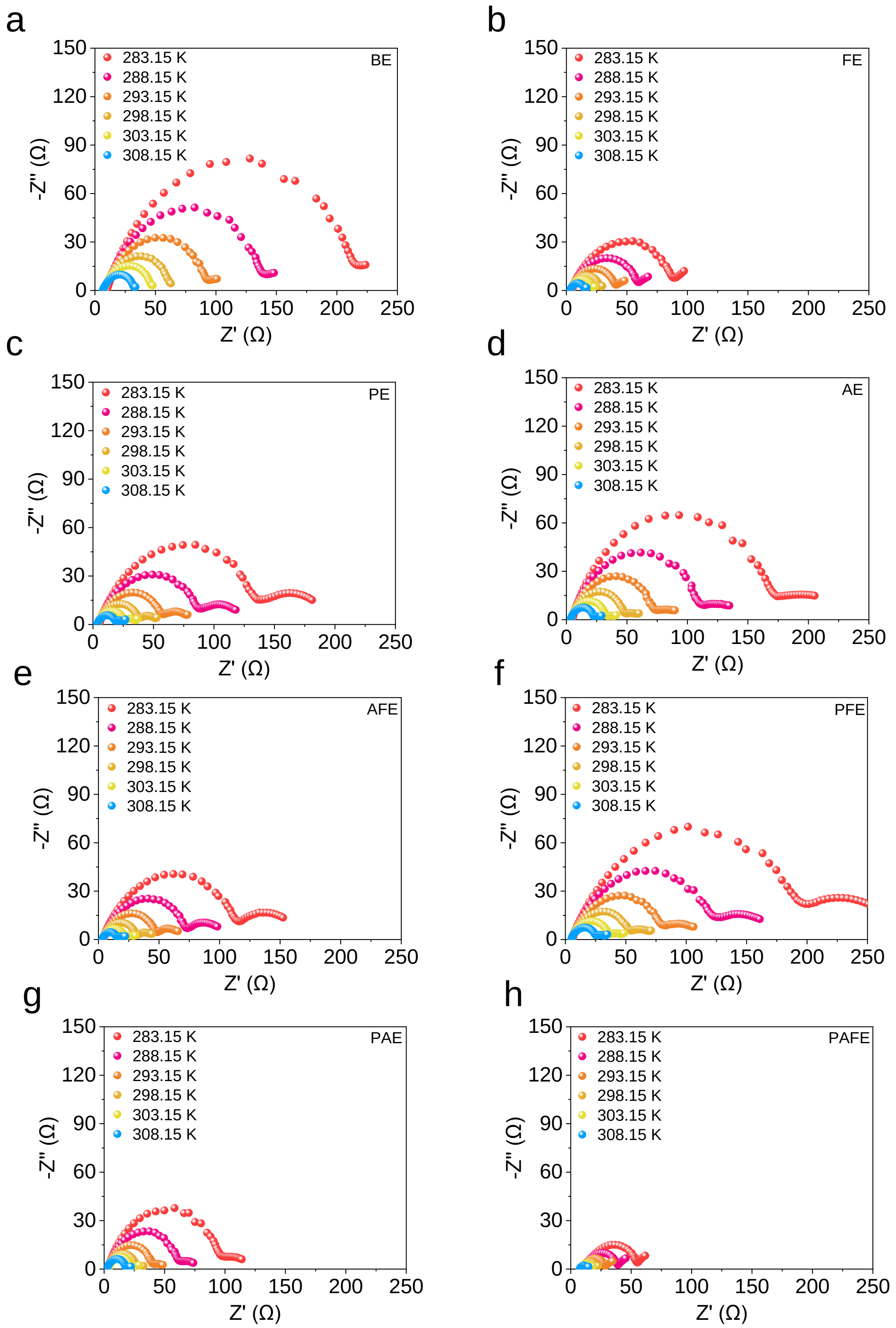

Figure S2. Nyquist plots at various temperatures for symmetric cells after forming stable SEI of BE (a), FE (b), PE (c), AE (d), AFE (e), PFE (f), PAE (g) and PAFE (h).

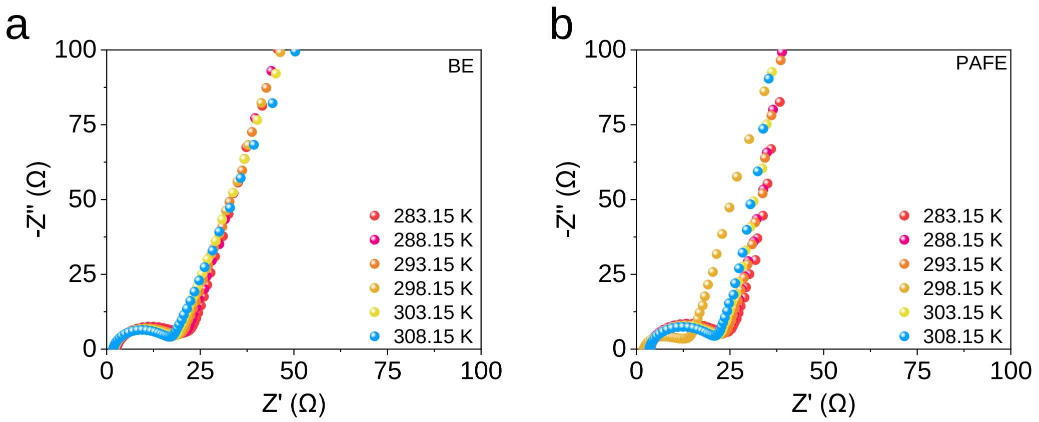

Figure S3. Nyquist plots at various temperatures for symmetric cells after forming stable CEI of BE (a) and PAFE (b).

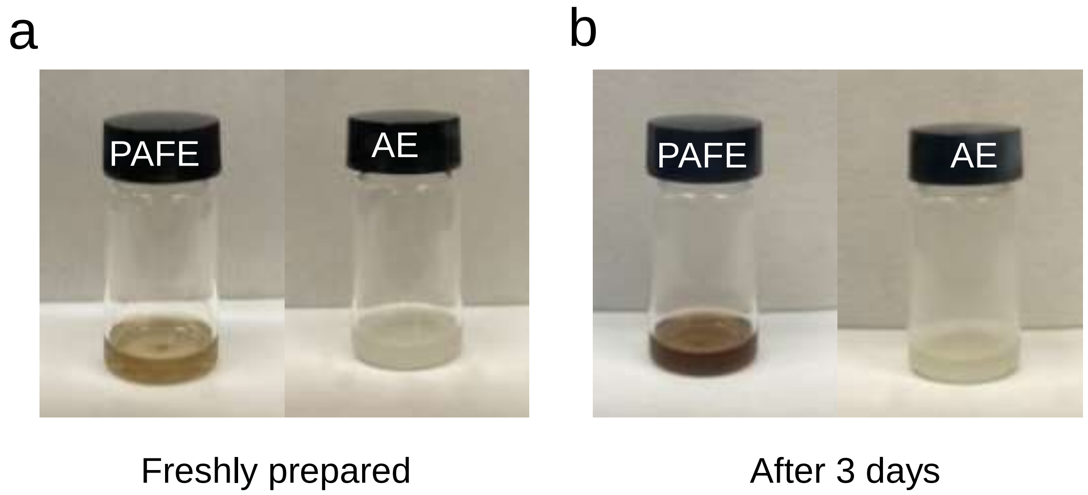

Figure S4. Digital photographs showing the colors of different electrolytes before (a) and after (b) standing at 30 ℃ for 3 days. All the electrolyte solutions were freshly prepared and stored in an Ar-filled glovebox.

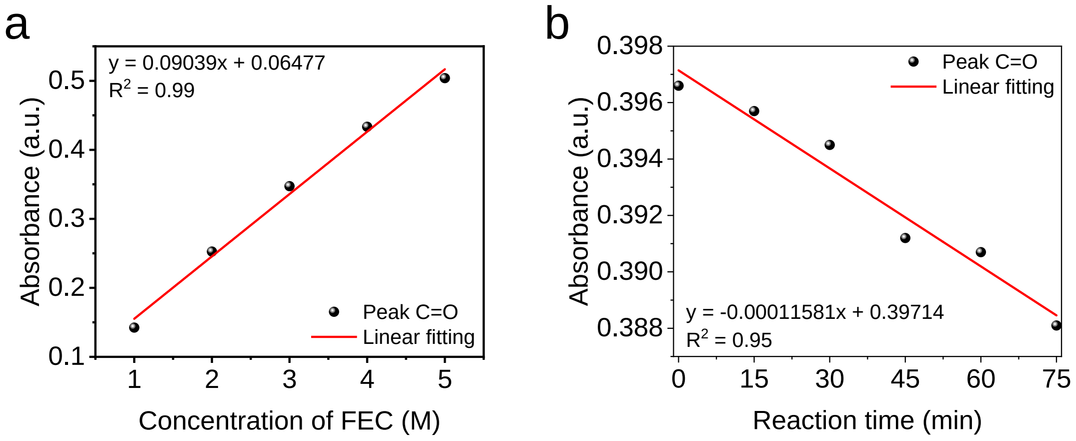

Figure S5. (a) FTIR spectroscopy-based calibration curve: FEC concentration vs. C=O characteristic peak absorbance. (b) FTIR spectroscopy-monitored temporal evolution of C=O peak intensity during reaction.

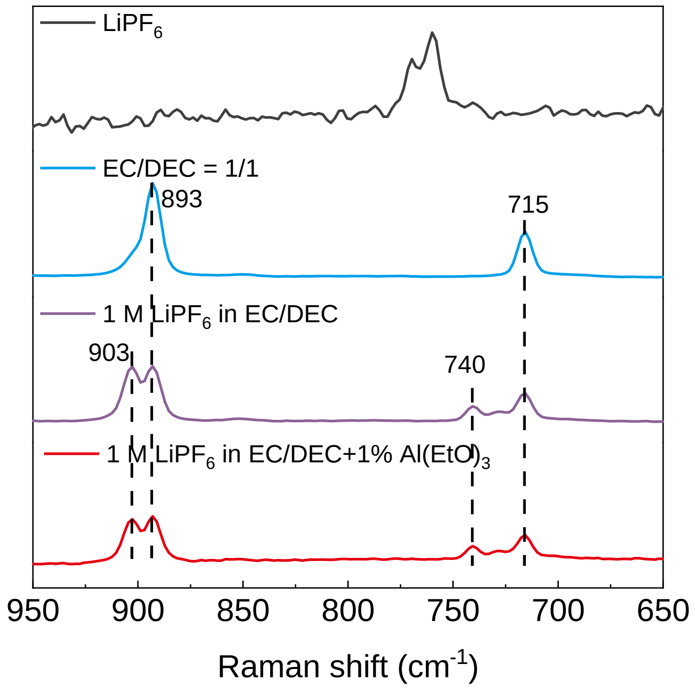

Figure S6. Enlarged Raman spectra of $LiPF_6$, ethylene carbonate (EC)/diethyl carbonate (DEC) solution (EC/DEC = 1/1 by volume), 1 M $LiPF_6$ dissolved in the EC/DEC = 1/1 (v/v) solution, and 1 M $LiPF_6$ and 1% $Al(EtO)_3$ dissolved in the EC/DEC = 1/1 (v/v) solution in the frequency range of 950–650 cm^{-1}.

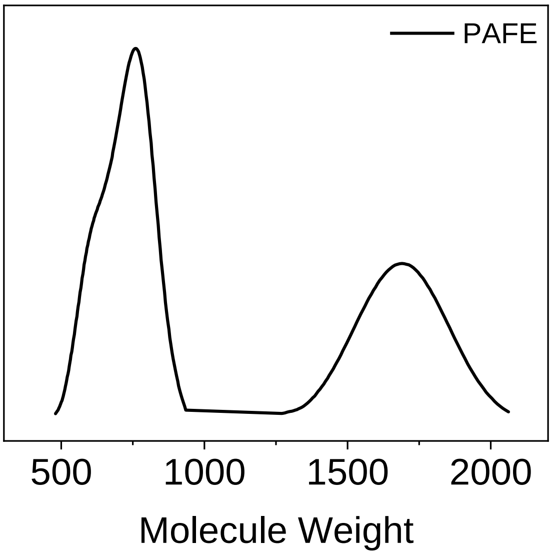

Figure S7. Molecular weight distribution map of macromolecular compounds generated by the reaction of three additives.

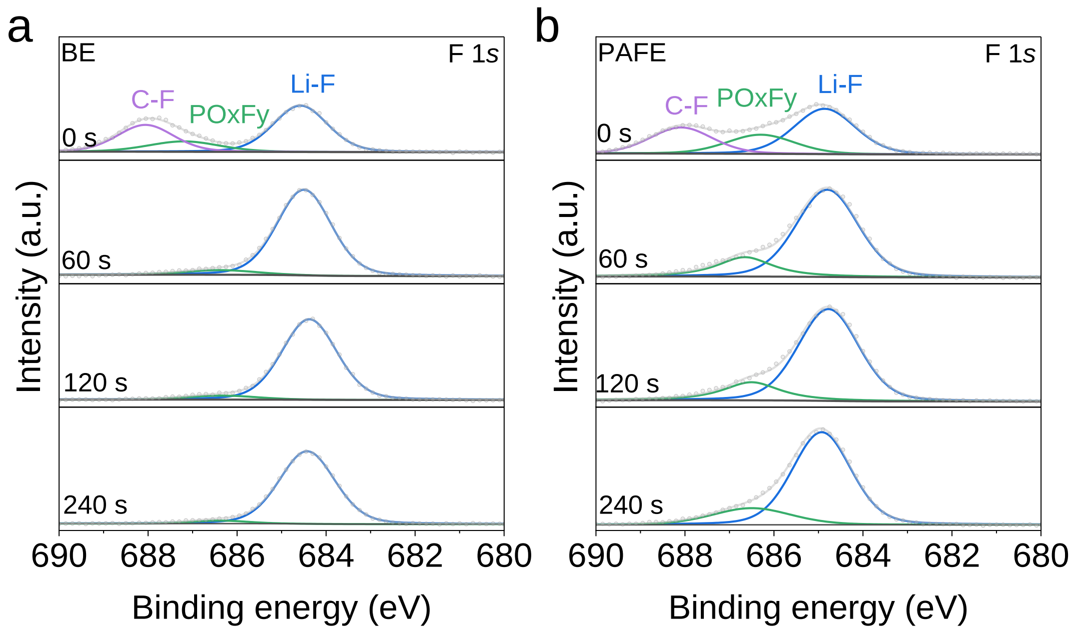

Figure S8. XPS analysis of lithium metal anode cycled in different electrolyte systems. XPS F 1*s* spectra of lithium metal anode in the BE (a) and the PAFE (b) after 10 cycles at the current density of 1 mA cm^{-2}.

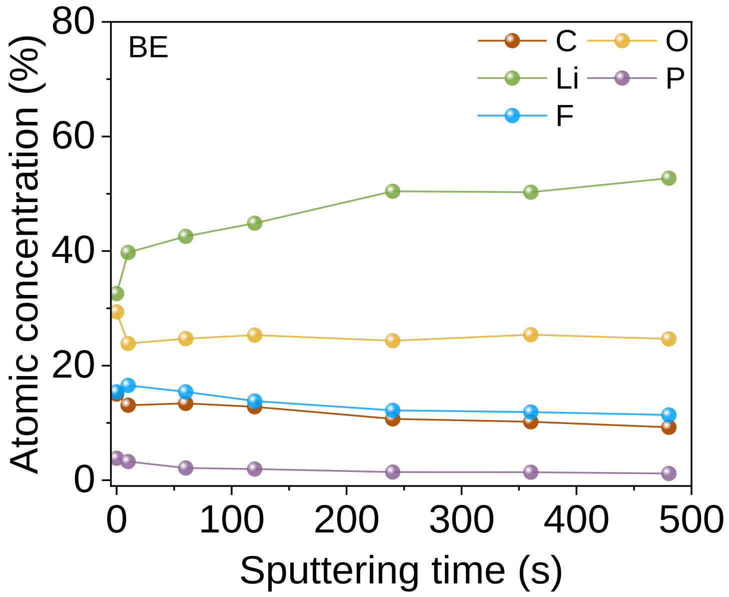

Figure S9. Atomic concentration of detected elements on SEI formed in BE.

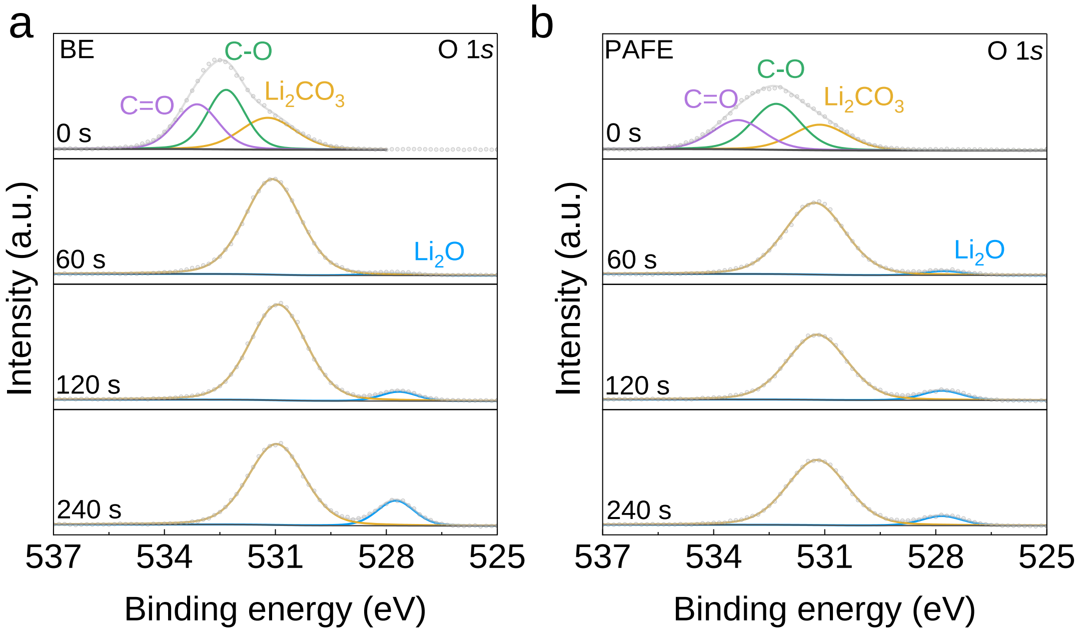

Figure S10. XPS analysis of lithium metal anode cycled in different electrolyte systems. XPS O 1*s* spectra of lithium metal anode in the BE (a) and the PAFE (b) after 10 cycles at the current density of 1 mA cm^{-2}.

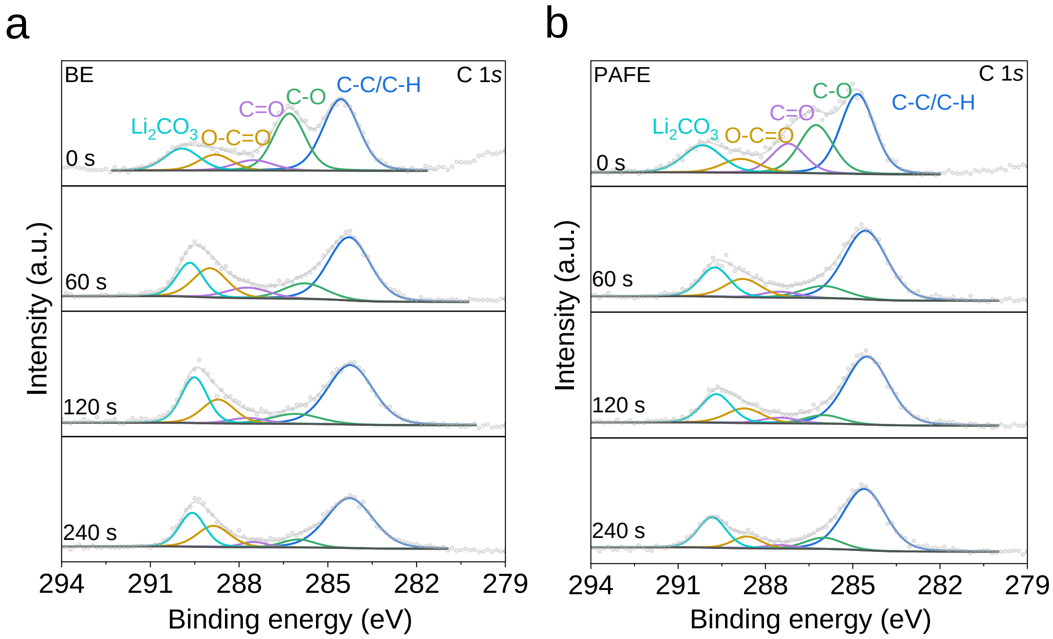

Figure S11. XPS analysis of lithium metal anode cycled in different electrolyte systems. XPS C 1*s* spectra of lithium metal anode in the BE (a) and the PAFE (b) after 10 cycles at the current density of 1 mA cm^{-2}.

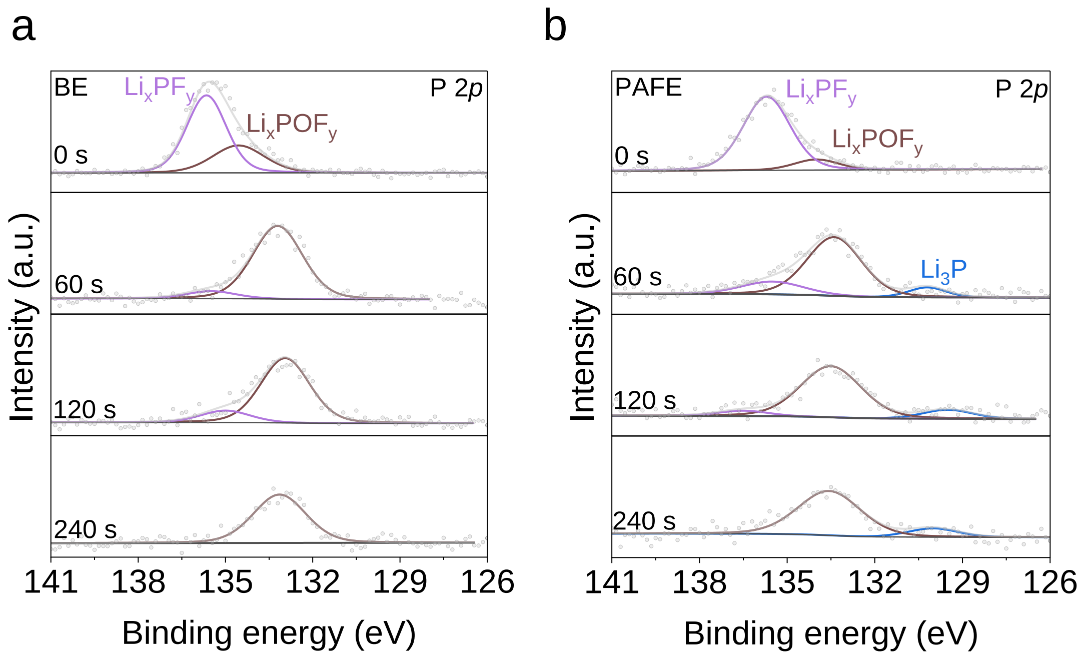

Figure S12. XPS analysis of lithium metal anode cycled in different electrolyte systems. XPS P 2*p* spectra of lithium metal anode in the BE (a) and the PAFE (b) after 10 cycles at the current density of 1 mA cm^{-2}.

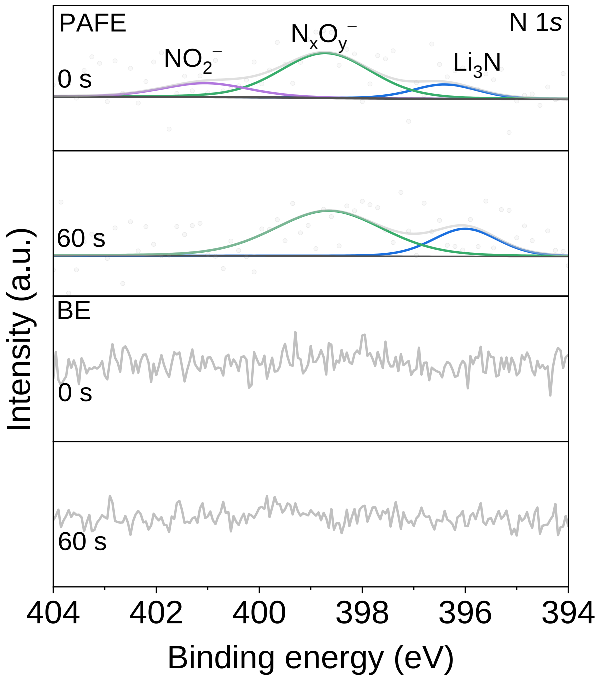

Figure S13. XPS N 1*s* spectra of lithium metal anode in the PAFE and the BE after 10 cycles at the current density of 1 mA cm^{-2}.

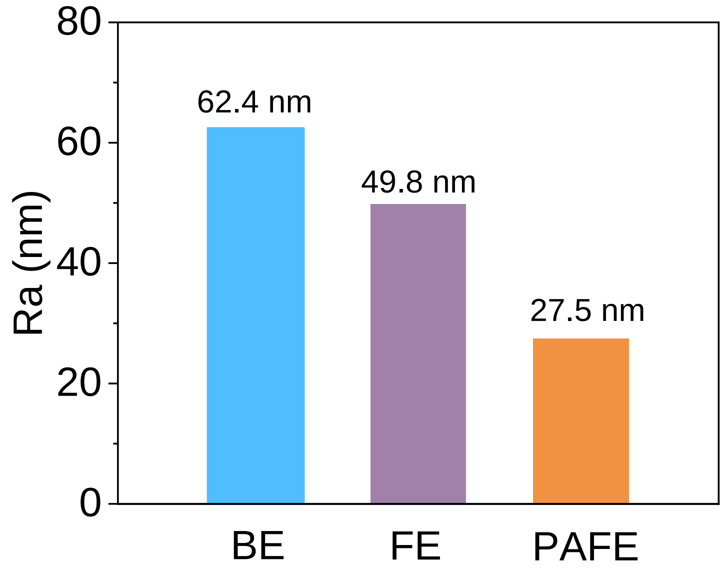

Figure S14. surface roughness (j) and force-displacement curves (k) of the deposited Li metal using BE, FE and PAFE at a current density of 0.5 mA cm^{-2} and a capacity of 3 mAh cm^{-2}.

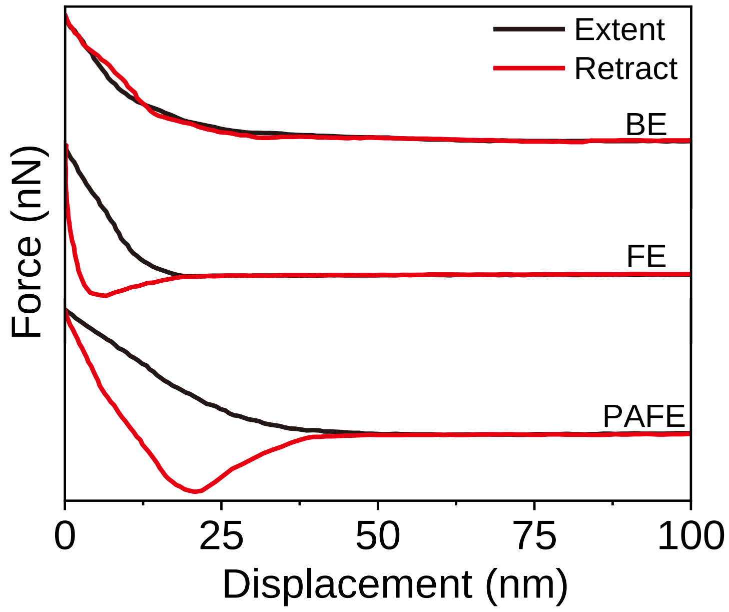

Figure S15. force-displacement curves of the deposited Li metal using BE, FE and PAFE at a current density of 0.5 mA cm^{-2} and a capacity of 3 mAh cm^{-2}. "Extend" and "Retract" indicate the direction of motion of the cantilever with respect to the sample.

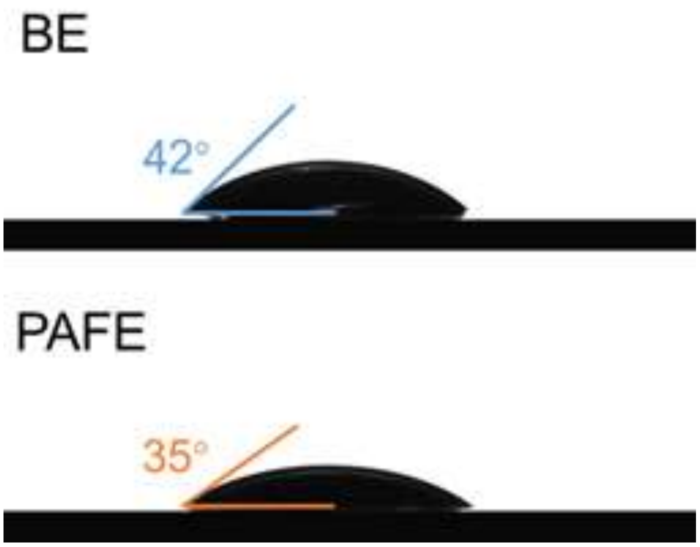

Figure S16. Contact angle of BE and PAFE with polypropylene separator.

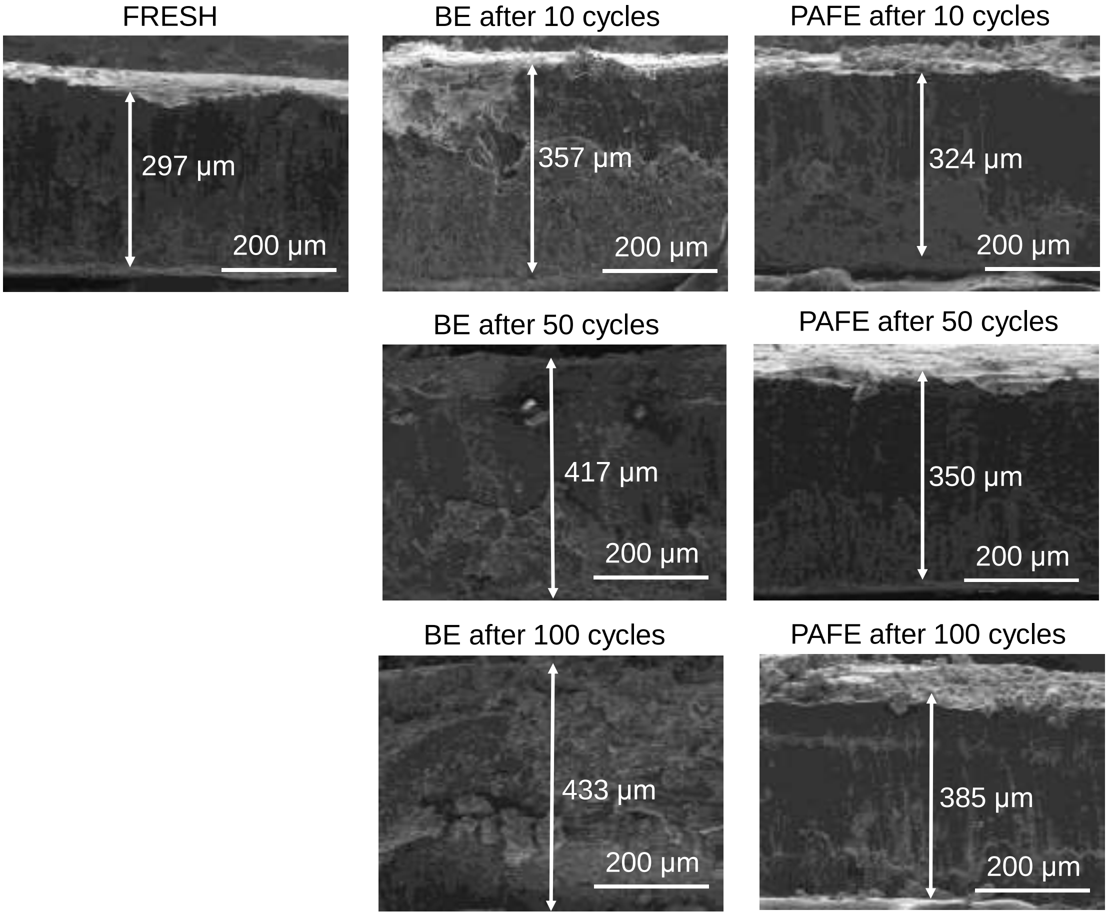

Figure S17. SEM images of the thickness evolution of Li metal anodes during long-term cycles in different electrolytes. The current density and capacity of Li||Li cells are 1 mA cm^{-2} and 1 mAh cm^{-2}, respectively.

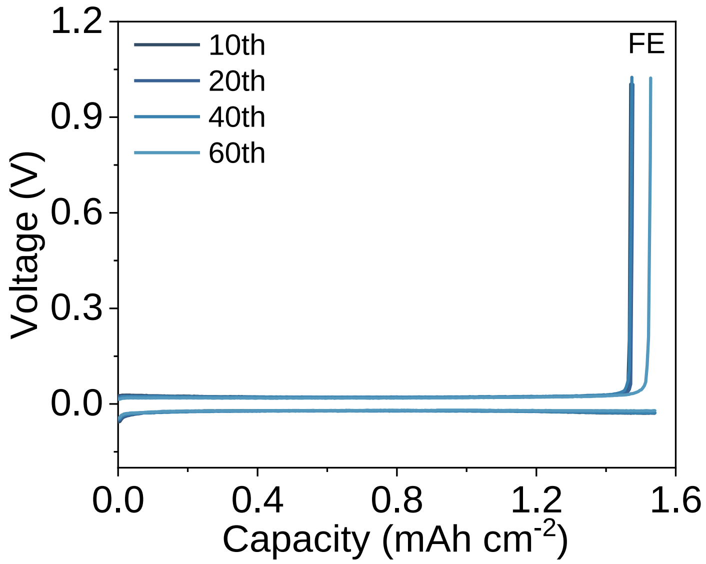

Figure S18. Voltage profiles of the Li||Cu cells with FE.

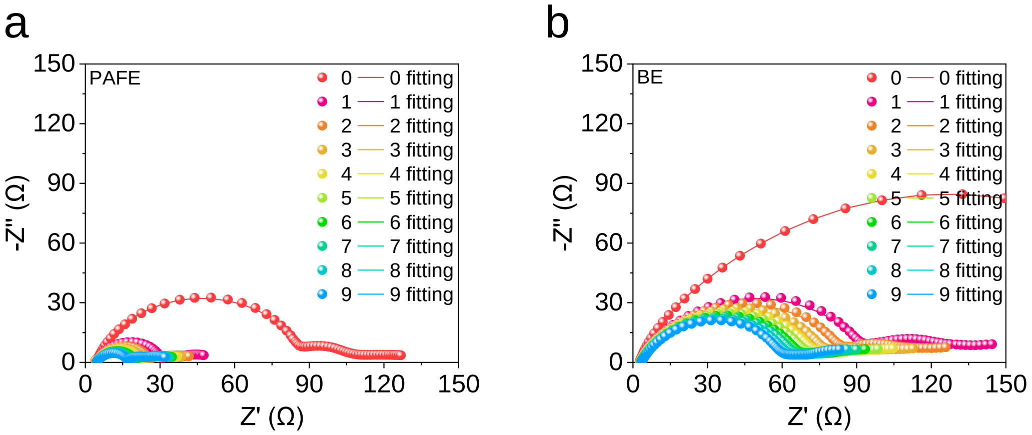

Figure S19. Electrochemical impedance spectra of the Li||Li cells using the PAFE (a) and BE (b) with an increasing cycle number (0-9 cycle). The current density is 1 mA cm^{-2} and the capacity is 1 mAh cm^{-2}.

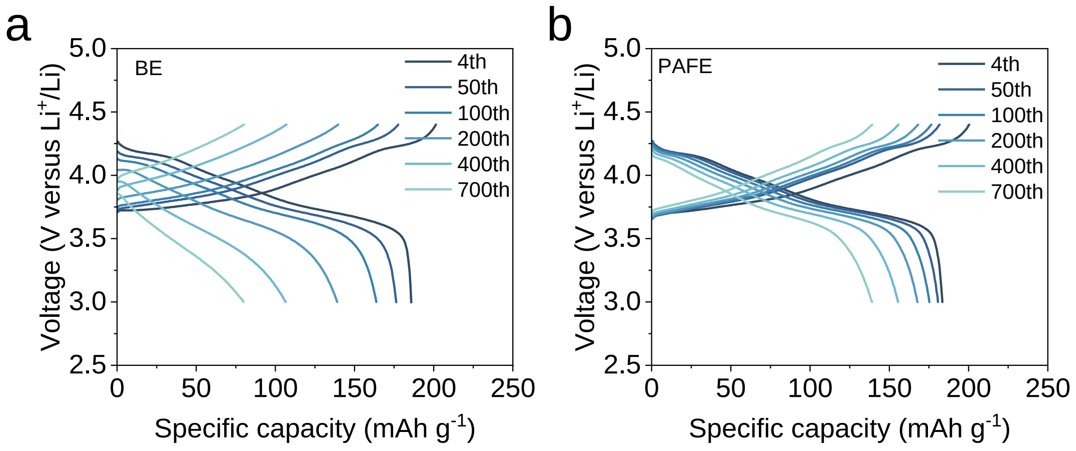

Figure S20. Corresponding voltage profiles of Li||NCM811 (0.4 mAh cm^{-2}) cells using the BE (a) and PAFE (b) at a 4.4 V cut-off voltage.

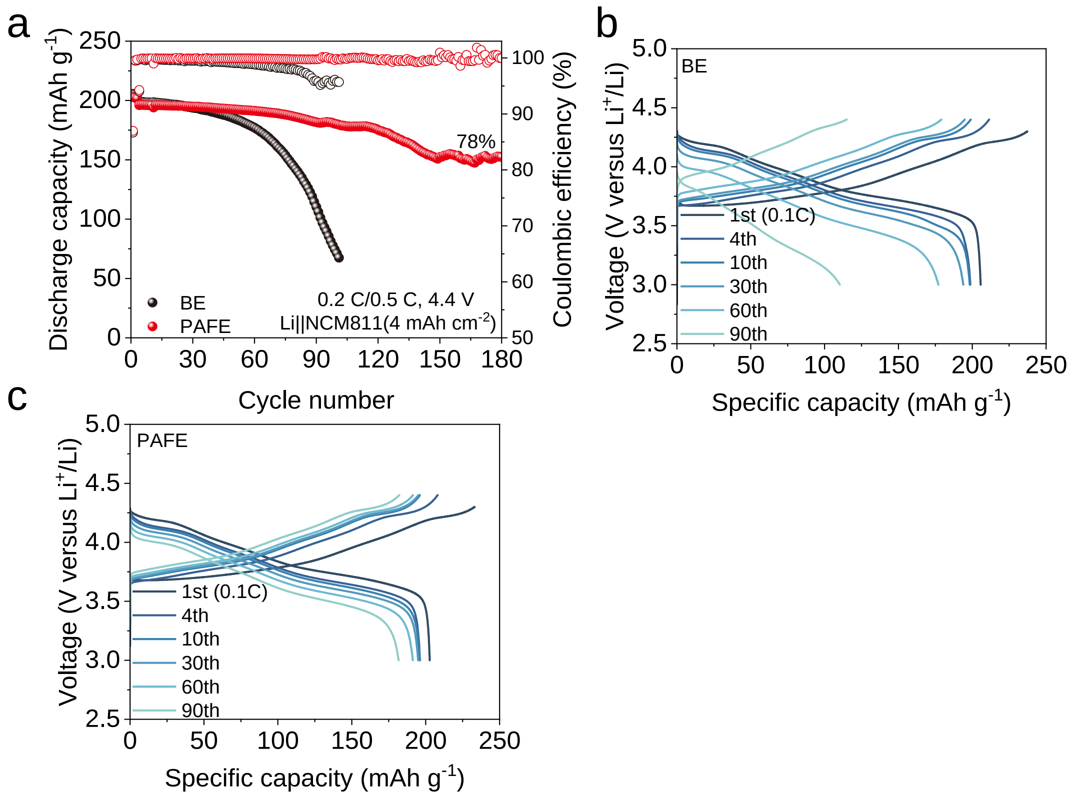

Figure S21. Cycling performance (a) and corresponding voltage profiles of Li||NCM811 (4 mAh cm^{-2}) cells using the BE (b) and PAFE (c) at a 4.4 V cut-off voltage.

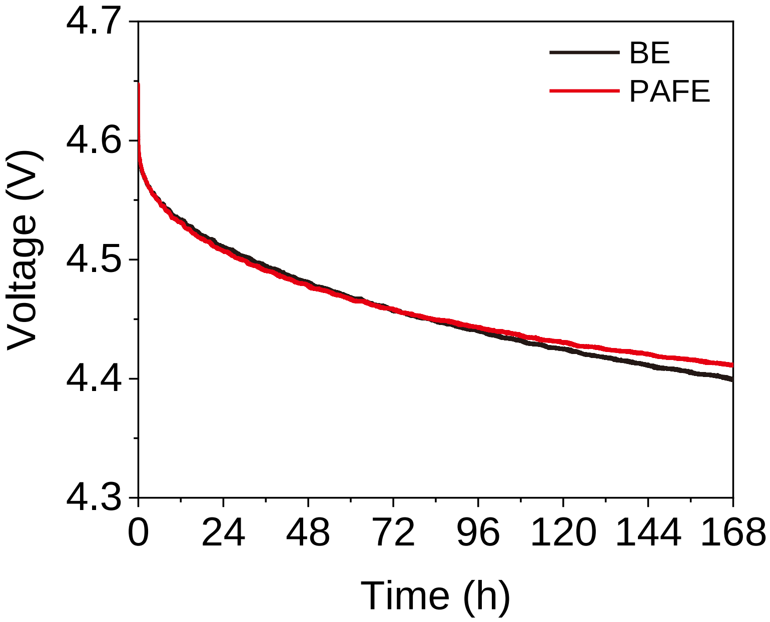

Figure S22. Voltage decay of Li||NCM811 cells using different electrolytes.

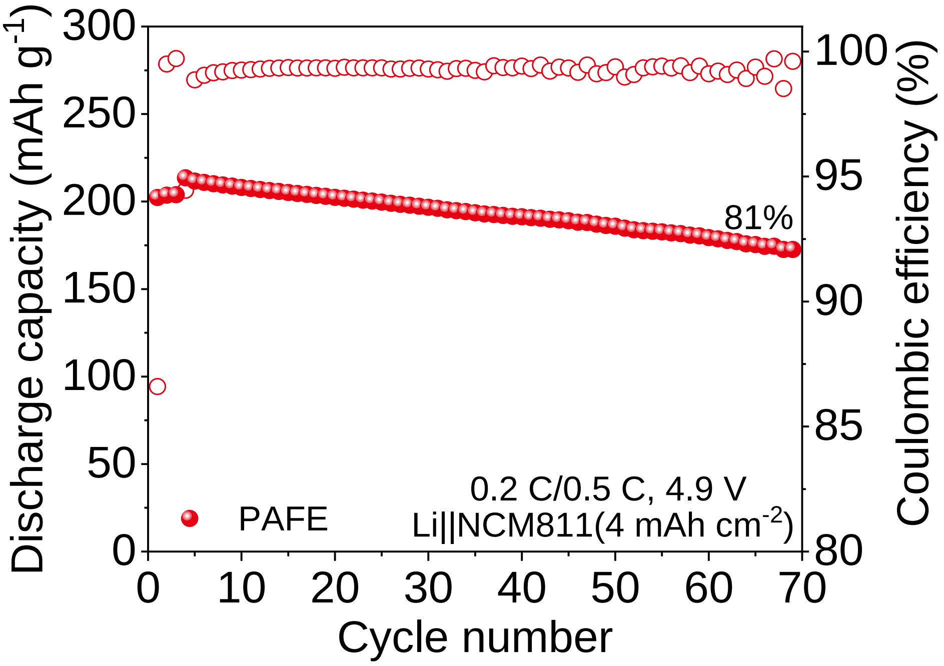

Figure S23. Cycling performance of the Li||NCM811 cell at 0.2 C charge/0.5 C discharge in the voltage range of 3.0–4.9 V.

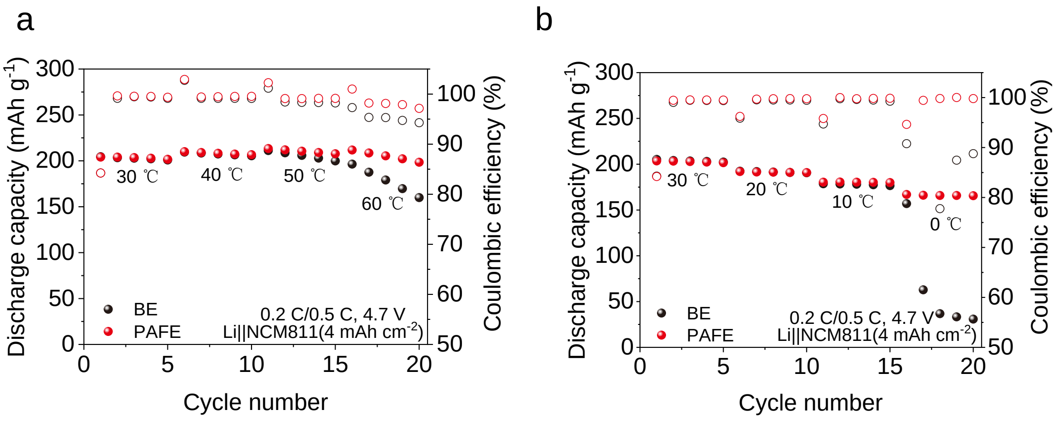

Figure S24. Cycling performance of Li||NCM811 cells using BE and PAFE electrolytes under high (a) and low (b) temperature conditions.

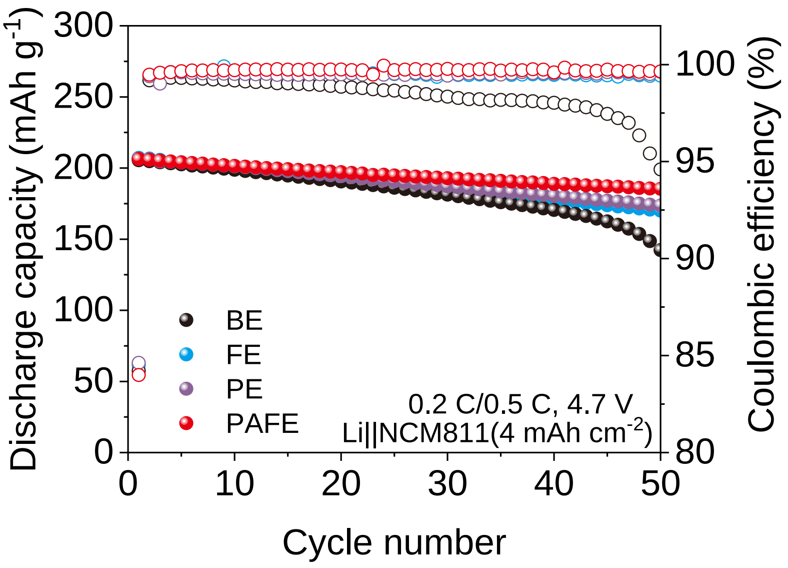

Figure S25. Electrochemical characterization of the Li||NCM811 cells. Cycling performance of the coin cells with BE, FE, PE and PAFE electrolytes at 0.2 C charge/0.5 C discharge in the voltage range of 3.0–4.7 V.

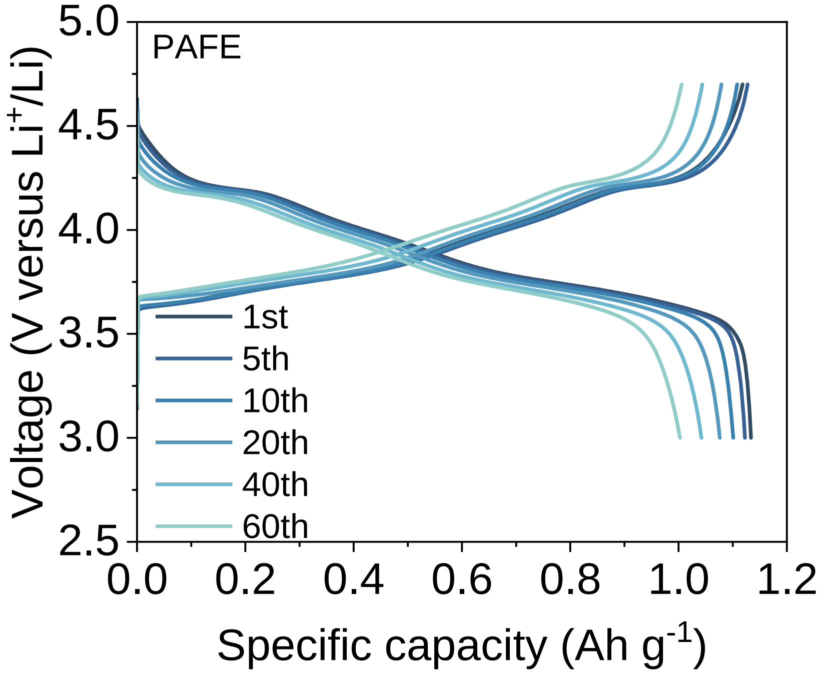

Figure S26. Corresponding voltage profiles of 1 Ah Li||NCM811 pouch cell using the PAFE at a 4.7 V cut-off voltage.

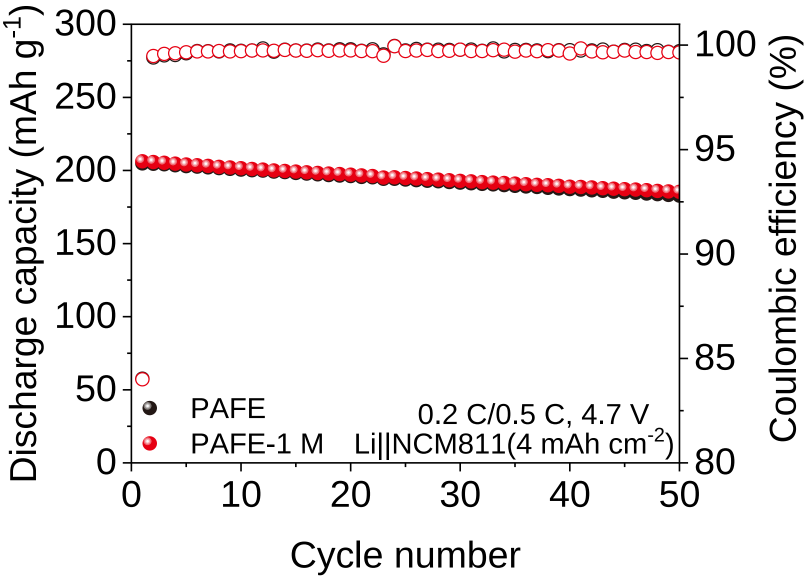

Figure S27. Cycling performance of Li||NCM811 cells with freshly prepared PAFE and PAFE stored for a month.

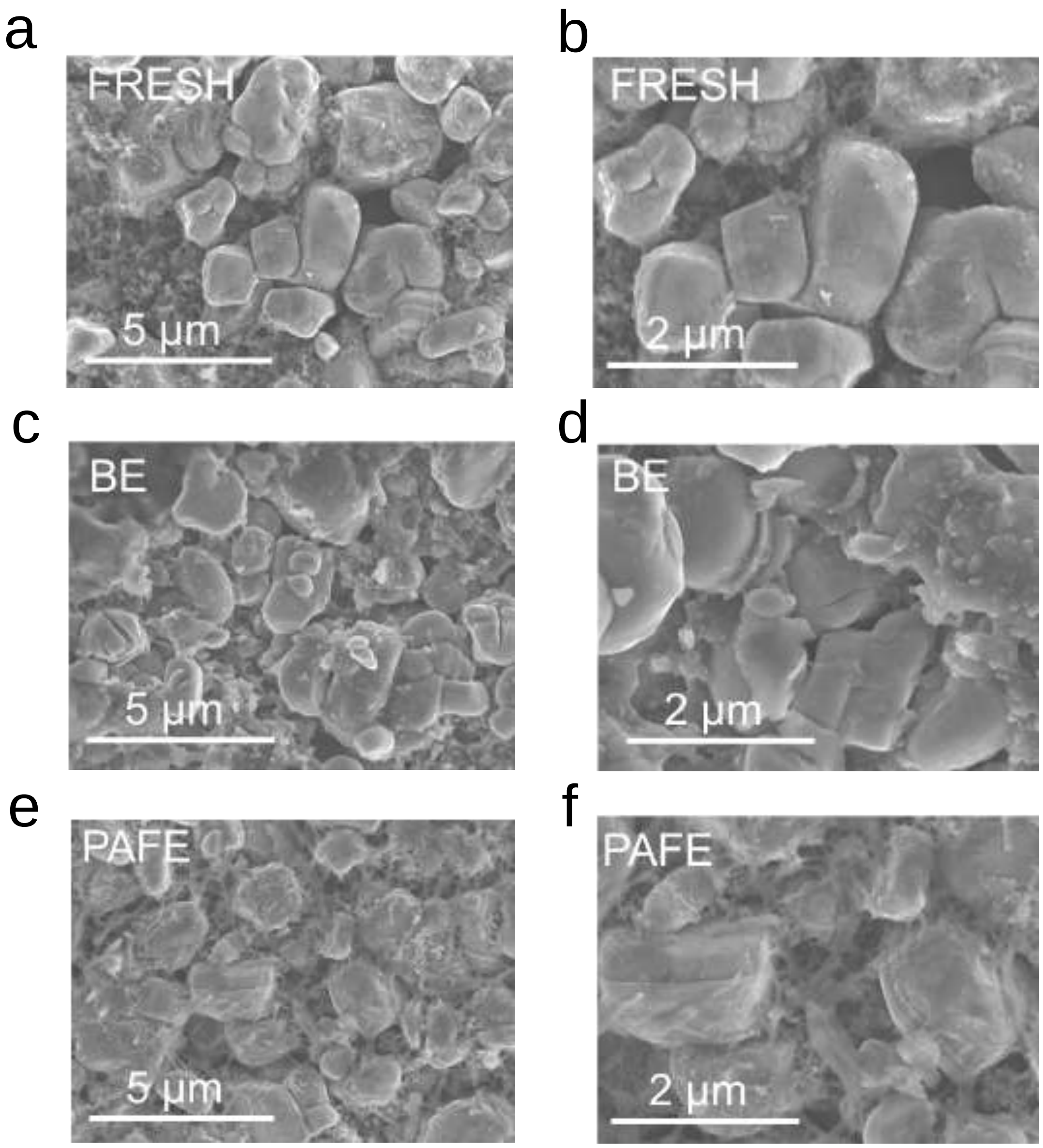

Figure S28. SEM images of NCM811 cathodes surface in the Fresh (a, b), BE (c, d) and PAFE (d, e). All the cells were cycling for 50 times at the specific current of 0.2C/0.5C. (1 C=200 mA g^{-1})

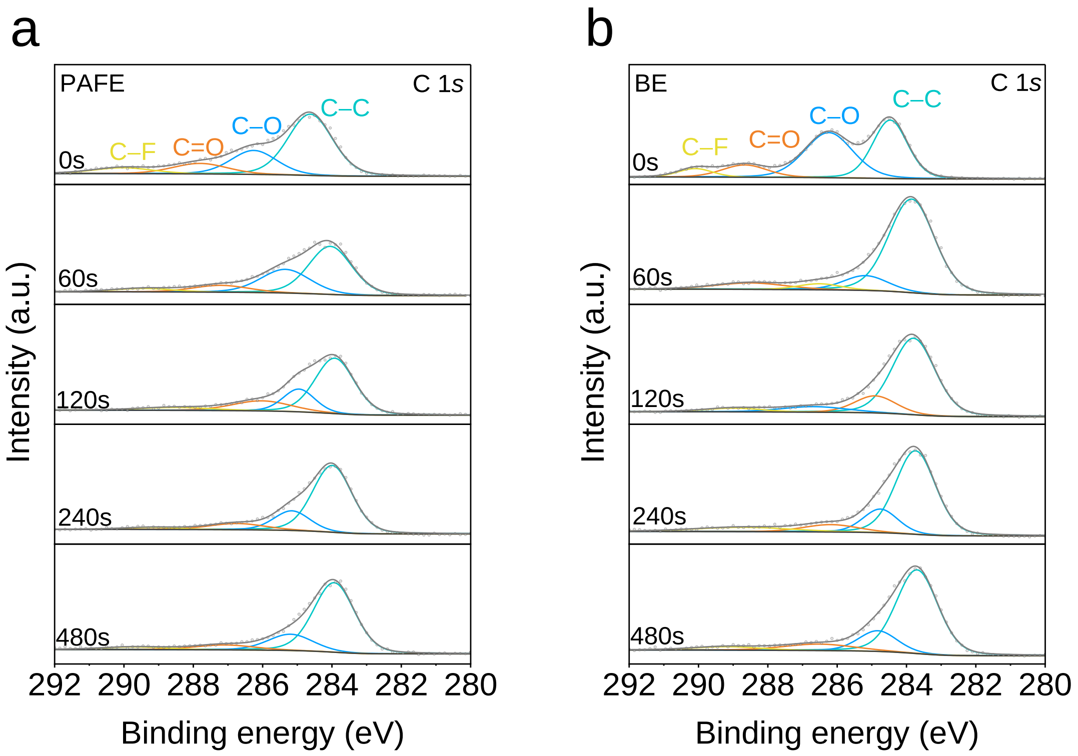

Figure S29. C 1*s* spectra of NCM811 cathodes in PAFE (a) and BE (b) after cycling for 50 times.

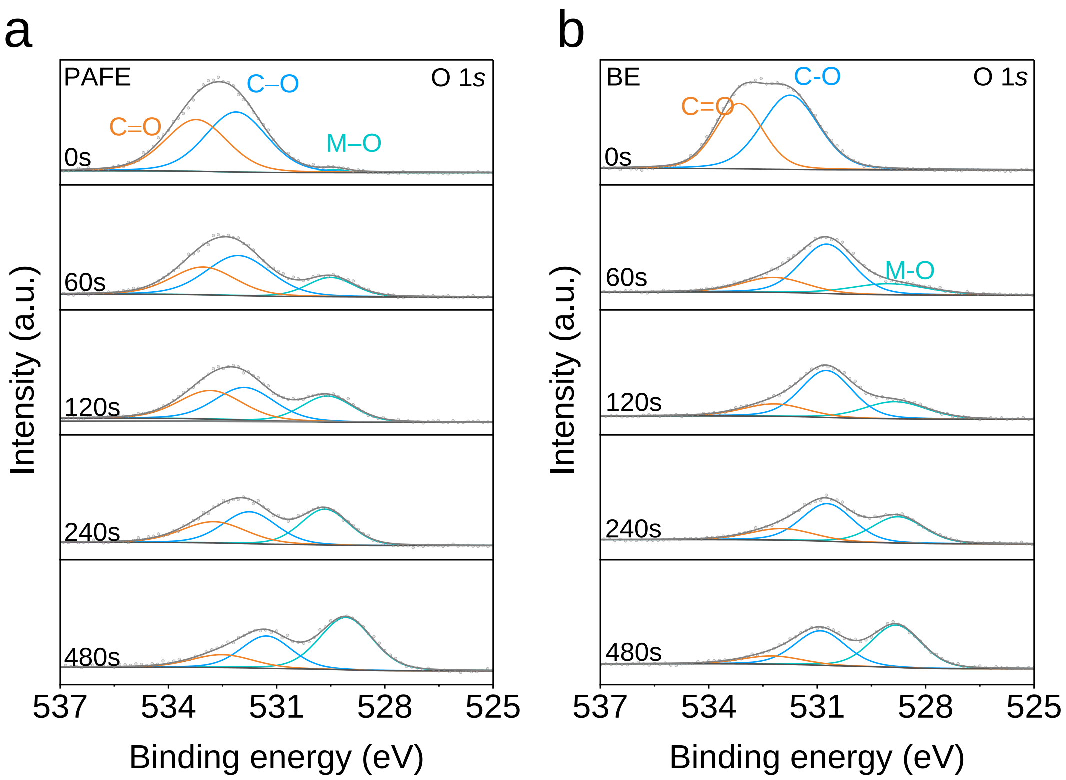

Figure S30. O 1*s* spectra of NCM811 cathode in PAFE (a) and BE (b) after cycling for 50 times.

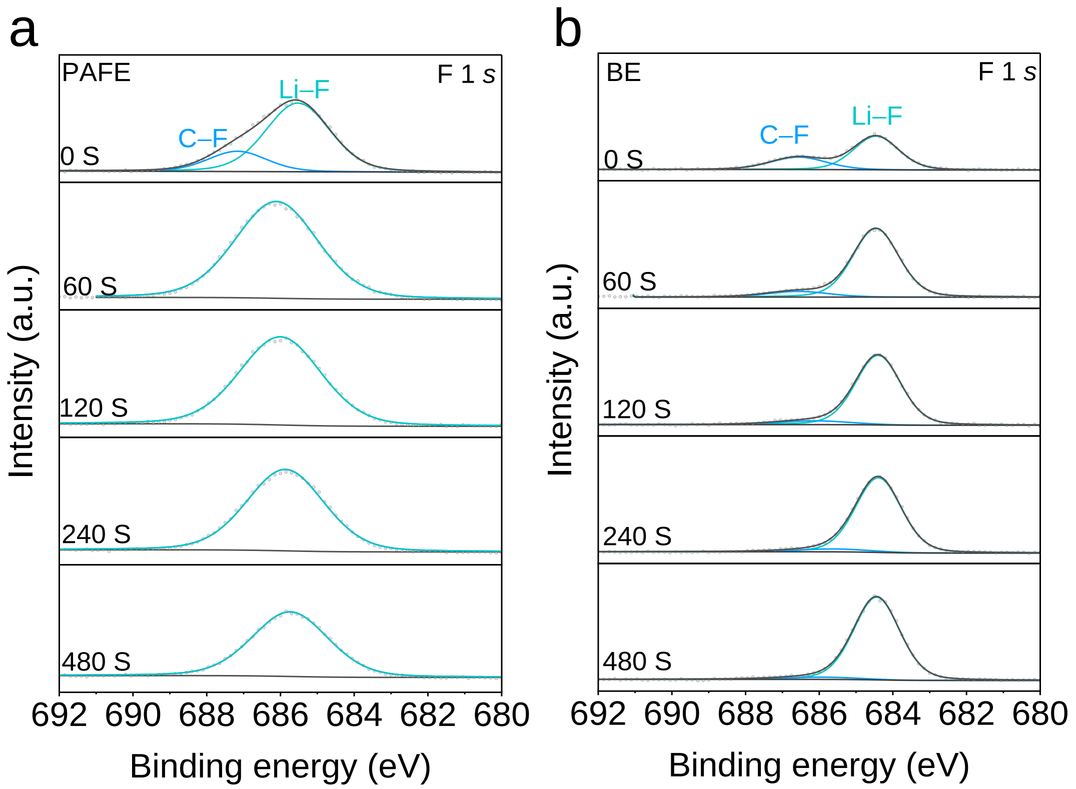

Figure S31. F 1*s* spectra of NCM811 cathode in PAFE (a) and BE (b) after cycling for 50 times.

Supplementary Tables

Table S1. R_{SEI} values of SEI at different temperatures and Arrhenius behavior of the reciprocal R_{SEI} and the calculated activation energy for Li diffusion through different SEI.

**Sample**	**Electrolyte composition**	**E_a(kJ mol^{-1})**
BE	1 M $LiPF_6$ in EC/DEC	61.1
FE	1 M $LiPF_6$ in EC/DEC + 20 vol.% FEC	60.9
PE	1 M $LiPF_6$ in EC/DEC + 10 vol.% PFPN	69.8
AE	1 M $LiPF_6$ in EC/DEC + 1 wt.% $Al(EtO)_3$	67.2
AFE	1 M $LiPF_6$ in EC/DEC + 1 wt.% $Al(EtO)_3$ + 20 vol.% FEC	73.2
PFE	1 M $LiPF_6$ in EC/DEC + 10 vol.% PFPN + 20 vol.% FEC	69.4
PAE	1 M $LiPF_6$ in EC/DEC + 10 vol.% PFPN + 1 wt.% $Al(EtO)_3$	67.4
PAFE	1 M $LiPF_6$ in EC/DEC + 10 vol.% PFPN + 1 wt.% $Al(EtO)_3$ + 20 vol.% FEC	48.5

Table S2. Ionic conductivity of electrolytes at 25 ℃. Five replicate measurements per sample are shown.

**Measurement**	**PAFE (mS cm^{-1})**	**BE (mS cm^{-1})**
1	7.02	8.09
2	7.17	8.17
3	7.02	8.2
4	6.97	8.16
5	6.94	8.16
Average	7.02	8.16

Table S3. Viscosity measurements of BE and PAFE electrolytes at 25 ℃. Ten data points were recorded during a single measurement (spindle speed: 250 rpm, total duration: 60 s).

**Time Point**	**BE (mPa s)**	**PAFE (mPa s)**
1	16.9	17.5
2	16.2	17.4
3	15.9	17.5
4	16.2	17.4
5	16.2	17.5
6	16.4	17.3
7	16.3	17.1
8	16.1	17.2
9	16.5	17.1
10	16.3	17
Average	16.3	17.3

Table S4. Comparison of our work with recent electrolyte works on Li||NCM811 coin cell.

Electrolyte	NCM811 mass loading (mg cm^{-2})	Cutoff voltage (V)	Non-flammability	Capacity retention (%)	Ref.
1 M $LiPF_6$ in EC/DEC/PFPN/FEC + 1 wt.% $Al(EtO)_3$	21.5	4.7	√	80% (140 cycles)	This work
1.2 M LiFSI in BMC + 0.75 wt.% $LiNO_3$ + 1wt.% LiDFBOP	24	4.4	√	80% (120 cycles)	Nat. Commun. **2024**, *15*, 3217
2 M LiFSI + 0.15 M LiDFP in DX/EGDBE	13	4.6	–	65% (130 cycles)	Angew. Chem. Int. Ed. **2024**, *63*, e202404109
1 M $LiPF_6$ in FEC/BTC/HFE/SL + 0.02M LiDFOB	1.88	4.7	–	82% (200 cycles)	Energy Environ. Sci. **2024**, *17*, 6113
1 M LiODFB in DGDE /MPN/FEC	5	4.5	–	78% (200 cycles)	Adv. Funct. Mater. **2024**, *34*, 2313319
2 M LiFSI in TFDMP	20	4.4	–	81% (200 cycles)	Nat. Commun. **2023**, *14*, 299
2 M LiFSI in BFE	18	4.4	–	90% (200 cycles)	Nat. Commun. **2023**, *14*, 1081
6.6 m LiFSI in DMEP/TTE + 5 vol.% PFPN	12	4.7	√	91% (100 cycles)	Adv. Mater. **2024**, *36*, 2312302
1 M $LiBF_4$ + 1 M LiDFOB in tFEP/FEC	22	4.6	–	80% (100 cycles)	Nat. Commun. **2023**, *14*, 1082

Table S5. The specifications of the Li||NCM811 pouch cell.

**Component of cell**	**Parameter**	**Value**
Cathode	Materials	NCM811
	Reversible capacity (mAh g^{-1})	200
	Active materials ratio (%)	94.5
	Areal capacity (mAh cm^{-2})	2.85
	Number of cathodes sheet	6
Anode	Materials	Li
	Areal capacity (mAh cm^{-2})	4.12
	Unilateral thickness (um)	20
Electrolyte	Injection mass (g)	2.48
	E/C ratio	2.19
Separator	Materials	PP
	Thickness (um)	16
Cell	Average discharge voltage (V)	3.87
	Capacity (Ah)	1.13
	Mass (g)	14.99
	Energy density (Wh kg^{-1})	292.7

Table S6. Lattice constant of NCM811 cathodes cycled in different electrolytes.

	a(Å)	c(Å)	c/a	Δ(%)
Fresh	2.87452	14.20294	4.940978	/
BE	2.83589	14.40906	5.080966	2.83
PAFE	2.86179	14.24716	4.978409	0.76
